# Thermal Manipulation During Embryogenesis Impacts H3K4me3 and H3K27me3 Histone Marks in Chicken Hypothalamus

**DOI:** 10.3389/fgene.2019.01207

**Published:** 2019-11-26

**Authors:** Sarah-Anne David, Anaïs Vitorino Carvalho, Coralie Gimonnet, Aurélien Brionne, Christelle Hennequet-Antier, Benoît Piégu, Sabine Crochet, Nathalie Couroussé, Thierry Bordeau, Yves Bigot, Anne Collin, Vincent Coustham

**Affiliations:** ^1^BOA, INRA, Université de Tours, Nouzilly, France; ^2^PRC, CNRS, IFCE, INRA, Université de Tours, Nouzilly, France

**Keywords:** thermal manipulation, epigenetic, reprogramming, histone post-translational modification, chicken

## Abstract

Changes in gene activity through epigenetic alterations induced by early environmental challenges during embryogenesis are known to impact the phenotype, health, and disease risk of animals. Learning how environmental cues translate into persisting epigenetic memory may open new doors to improve robustness and resilience of developing animals. It has previously been shown that the heat tolerance of male broiler chickens was improved by cyclically elevating egg incubation temperature. The embryonic thermal manipulation enhanced gene expression response in muscle (*P. major*) when animals were heat challenged at slaughter age, 35 days post-hatch. However, the molecular mechanisms underlying this phenomenon remain unknown. Here, we investigated the genome-wide distribution, in hypothalamus and muscle tissues, of two histone post-translational modifications, H3K4me3 and H3K27me3, known to contribute to environmental memory in eukaryotes. We found 785 H3K4me3 and 148 H3K27me3 differential peaks in the hypothalamus, encompassing genes involved in neurodevelopmental, metabolic, and gene regulation functions. Interestingly, few differences were identified in the muscle tissue for which differential gene expression was previously described. These results demonstrate that the response to embryonic thermal manipulation (TM) in chicken is mediated, at least in part, by epigenetic changes in the hypothalamus that may contribute to the later-life thermal acclimation.

## Introduction

During the last several decades, it became clear that the epigenome of eukaryotes dynamically responds to the environment. Stress, diet, behavior, toxins, and other factors modulate gene expression through epigenetic modifications such as chemical additions to DNA and histones that can be stably maintained during mitotic divisions and do not change the primary DNA sequence (Fresard et al., 2013; David et al., 2017a). Early embryogenesis is a critical window of sensitivity to the environment, and environmentally induced changes can be transmitted during development by subsequent cell divisions. Among the best-studied examples are the persistent epigenetic differences in humans associated with prenatal exposure to famine during the Dutch hunger winter (Heijmans et al., 2008) and the maternal diet supplemented with folate that induces pseudo-agouti phenotypes in mice (Feil and Fraga, 2012). Early temperature exposure is also known to affect the phenotypes of plants and animals through epigenetic reprogramming. In yeast, histone post-translational modifications, and in particular H3K4 methylation, have emerged as primary actors in the mitotic memory of the environmental stress response (Fabrizio et al., 2019). In plants, the molecular basis of vernalization (the acceleration of flowering by prolonged cold) has been extensively studied and notably involves the quantitative accumulation of trimethylation of histone H3 at lysine 27 (H3K27me3) during cold periods (Coustham et al., 2012). Heat acclimation-mediated cross-tolerance resulting from the enhancement of innate cytoprotective pathways in rats was shown to involve epigenetic mechanisms such as post-translational histone modification and altered levels of chromatin modifiers during the acclimation phase (Horowitz, 2017). In chickens, post-natal heat acclimation during the 3^rd^or 5^th^day of life was shown to improve temperature tolerance at 10 days of age through epigenetic changes such as DNA methylation at *BDNF* and histone post-translational modifications at *BDNF*, *Eif2b5* and *HSP70* in the hypothalamus (Yossifoff et al., 2008; Kisliouk et al., 2010; Kisliouk et al., 2011; Kisliouk et al., 2017).

Broiler chickens have long been selected for growth performance traits, with significant progress in genetic selection during the last several decades. However, the huge improvement in chicken body weight and muscle growth has not been associated with a similar increase in visceral organs such as heart and lungs necessary for hyperventilation and heat loss (Havenstein et al., 2003). As a consequence, chickens have became less able to cope with extreme environmental temperatures, as illustrated by the 2003 hot spells in France that resulted in the death of several millions of meat-type chickens (Yahav and McMurtry, 2001; St-Pierre et al., 2003). In order to improve bird thermal tolerance and welfare without impairing growth in later life, a protocol of early exposure to heat during egg embryonic development has been proposed (Piestun et al., 2008). The TM treatment during embryogenesis consists in increasing egg incubation temperature to 39.5°C for 12 h/d, in contrast to a continuous incubation at 37.8°C under standard condition, during a time-window that coincides with the development of the hypothalamic-pituitary-thyroid axis (between E7 and E16; chicken eggs hatch around E21) (Piestun et al., 2008; Loyau et al., 2013). The TM treatment, that can easily be applied by hatcheries, was shown to modify several physiological parameters such as lowering the internal temperature and to improve heat tolerance of broilers during acute heat stress at slaughter age (5 weeks old) with little effect on animal performance (Piestun et al., 2008; Loyau et al., 2013).

To investigate the impact of TM at the molecular level, a muscle transcriptome analysis was performed on TM and control (C) chickens that were either heat challenged or not challenged before sampling at 5 weeks of age (Loyau et al., 2016). Only a few genes (28) were found to be differentially expressed (DE) between C and TM. Interestingly, 759 genes were found to be DE between the heat-challenged TM (32°C for 5h at 35 days of age) and TM individuals that were left at thermally neutral condition (21°C) while only 128 were found to be DE in the comparison between heat-challenged controls and controls left in thermally neutral conditions (Loyau et al., 2016). Hence, a number of genes may have been conditioned to respond to further heat exposure by a mechanism that may involve epigenetic changes. In addition, the analysis showed that 26 genes involved in chromatin organization, remodeling, and gene silencing were DE when TM chickens were exposed to a heat challenge (vs. TM in thermal neutral conditions) (Loyau et al., 2016). We therefore hypothesized that an epigenetic reprogramming induced by the embryonic heat exposure may have modified part of the chromatin landscape thus affecting gene expression in favor of heat acclimation. To test this hypothesis, we performed a whole-genome analysis of H3K4me3 and H3K27me3 histone marks. Both *P. major* muscle and hypothalamus tissues of TM and C chickens were investigated because of the transcriptome changes previously shown and the involvement of brain in postnatal acclimation respectively (Yossifoff et al., 2008). Here, we show that embryonic TM affected H3K4me3 and, to a lesser extent, H3K27me3 in the hypothalamus of 35-day-old chickens whereas the effects were very limited in the muscle. Most differential peaks occurred within the 5’ part of genes that were involved in biological functions relevant to neurodevelopmental, metabolic, and gene regulation functions. This study showed that several genes that code for components of the corticotropin-releasing hormone (CRH) signaling pathway were affected at the histone level, suggesting that TM may affect biological processes similar to those affected by the postnatal acclimation model.

## Results

### TM Has a Long-Lasting Impact on the Epigenome of Developing Chickens

TM was applied (or not, for controls) on Cob500 eggs and the reproducibility of the embryonic treatment was compared to previous experiments by measuring cloacal temperature at hatching, as this has been shown to be decreased in TM chickens (Loyau et al., 2015). As expected, we found that cloacal temperature of male TM animals was significantly reduced at hatching (38.7°C in average, n = 54) compared to male controls (38.9°C, n = 68; p-value < 0.05). We then sampled hypothalamus and muscle samples of 35-day-old male TM and C chickens. ChIP followed by Illumina sequencing was performed using antibodies specific to H3K4me3 and H3K27me3 modifications. For hypothalamus samples, ∼23 to 75 thousands of peaks were obtained (**Table 1** and **Supplementary Table S1**). As expected, H3K27me3 peaks were broader than H3K4me3 peaks (**Supplementary Figure S1** and **Table S1**). 785 and 148 differential peaks (DP) were identified for H3K4me3 and H3K27me3 respectively in this tissue (**Table 1**). By contrast, only 45 and 20 peaks were found to be DE for H3K4me3 and H3K27me3 respectively in the muscle, representing less than 0.1% of detected peaks. A majority of DP showed reduced signal in TM animals for both marks and tissues (**Table 1**).

**Table 1:** Summary of ChIP-seq results.Three biological replicates were sequenced per tissue and mark.

	Hypothalamus		Muscle P. major	
	**H3K4me3**	**H3K27me3**	**H3K4me3**	**H3K27me3**
Analyzed peaks	30597	23098	75943	26562
Number of DP	785	148	45	20
% DP	2.57	0.64	0.06	0.08
Number of DP up	147	37	7	4
Number of DP down	638	111	38	16
Genes containing DP	975	69	44	17

Reads were mapped against the chicken genome Galgal5, and peaks were detected using PePr for H3K4me3 and epic for H3K27me3. DP, Differential Peaks. Comparisons (DP up, DP down) were calculated as TM vs. control.

**Figure 1:**
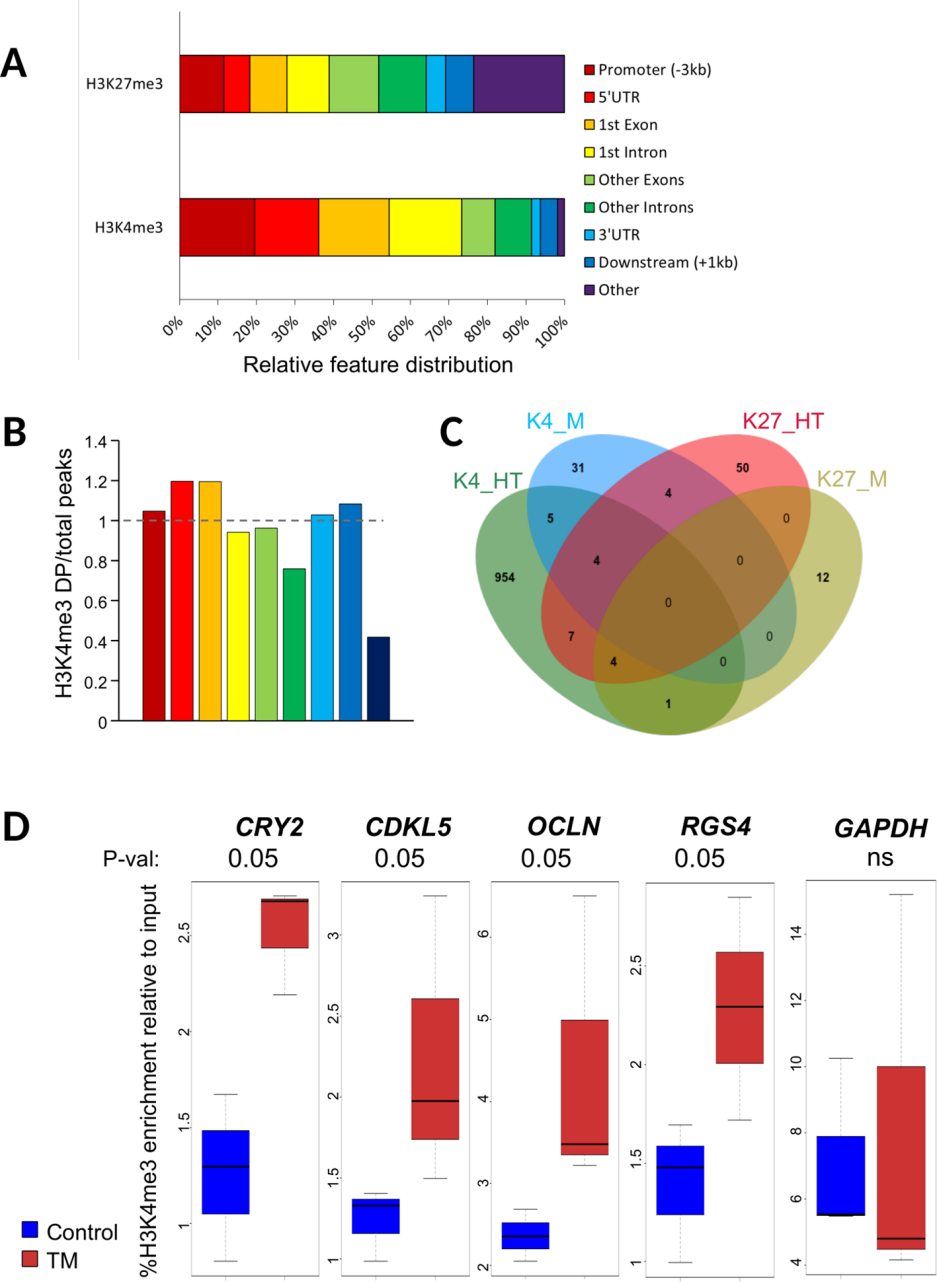
Characteristics of differential peaks. **(A)** Percentage of ChIP-seq H3K4me3 differential peaks across genomic features in hypothalamus relative to all differential peaks, obtained using GenomeFeatures. Promoter and downstream regions were defined upstream of Transcription Start and End Sites (TSS and TES, respectively), respectively. **(B)** Barplot showing the ratio for each feature between the percentage of differential peaks (DP) per feature relative to all DP and the percentage of all peaks per feature vs. all peaks for H3K4me3 in hypothalamus. The color legend is the same as in **Figure 1A**. **(C)** Venn diagram showing the number of genes containing a DP that are specific or common for H3K4me3 (K4) and H3K27me3 (K27) marks in hypothalamus (HT) and muscle (M) tissues. **(D)** Box plot of H3K4me3 relative enrichment to input at *CRY2*, *CDKL5*, *OCLN*, and *RGS4* loci in Control (blue) and TM (red) replicates. Box boundaries represent the first and third quartiles. The median is indicated by the bold horizontal line dividing the interquartile range. Upper and lower ticks indicate the 10th and 90th percentiles. Corresponding p-values are indicated at the top of each graph

We next investigated the distribution of DP. Hypothalamic H3K4me3 DP were found preferentially in genes, near the transcription start sites (TSS), in the first exon and in the first intron (**Figure 1A**). This result is consistent with genome-wide distribution of this mark in all the H3K4me3 peaks detected in the study (**Figure 1B**). Only DP outside of genes appeared underrepresented suggesting that H3K4me3 changes in the hypothalamus of TM animals occurred preferentially within genes. However, H3K27me3 hypothalamus DP were evenly distributed within the body of genes (**Figure 1A**), likely reflecting the broader size of peaks. However, one quarter of hypothalamus H3K27me3 DP were found outside of genes, which is similar to the genome-wide distribution of this mark in our experiment (**Supplementary Figure S2A**). Muscle distribution of DP was roughly similar to the hypothalamus, with the exception of the H3K4me3 distribution that appeared less skewed toward the 5’ end of genes (**Supplementary Figure S2B**).

We next sought to identify DP that were localized within genes (DP genes). For the hypothalamus, we found 975 genes containing at least one H3K4me3 DP (95% unique) and 69 genes containing at least one H3K27me3 DP. Muscle DP were found only in 44 and 17 genes for H3K4me3 and H3K27me3, respectively (**Table 1**). Overall, 25 DP genes (2.5%) were found in at least two conditions (shared between marks and/or tissues; **Figure 1C** and **Table S2**). Notably, eight genes including the *Protocadherin alpha-2*(*PCDHA2*) subunit involved in the establishment and function of specific cell to cell connections in the brain and seven nonannotated genes contained DP in three conditions (**Supplementary Table S2**). Moreover, 17 genes contained DP in two conditions, either in a tissue specific or a mark specific fashion with the exception of another protocadherin (*PCDHA11*) that contained a H3K4me3 DP in the hypothalamus and a H3K27me3 DP in muscle (**Supplementary Table S2**). The complete list of DP genes is provided in **Supplementary Table S2**.

To validate the ChIP-seq data, we performed ChIP-qPCR on biological replicates from the same experiment on a set of H3K4me3 DP in the hypothalamus. Primers were designed within or encompassing DP regions at *CCNF* (*cyclin F*), *CRY2* (*Cryptochrome 2*), *CDKL5* (*Cyclin Dependent Kinase Like 5*), *OCLN* (*Occludin*), and *RGS4* (*Regulator Of G Protein Signaling 4*) with an increase in TM condition. A nondifferential control at the *GAPDH* locus was included in the analysis. We found that H3K4me3 levels were significantly increased in all regions in TM hypothalamus samples from biological replicates thus confirming ChIP-seq data except for *CCNF* that only showed a nonsignificant trend toward an increase (**Figure 1D** and data not shown). To investigate the relationship between DP and gene expression, we also checked the expression of *CCNF*, *CDKL5*, *CRY2*, and *OCLN* by RT-qPCR. However, none of these appeared to be altered by the presence of DP as no transcript level was found significantly altered (**Supplementary Figure S4**).

### Differential Peaks Were Found Preferentially in Neurodevelopmental, Metabolic and Transcriptional Regulator Genes

**Figure 2:**
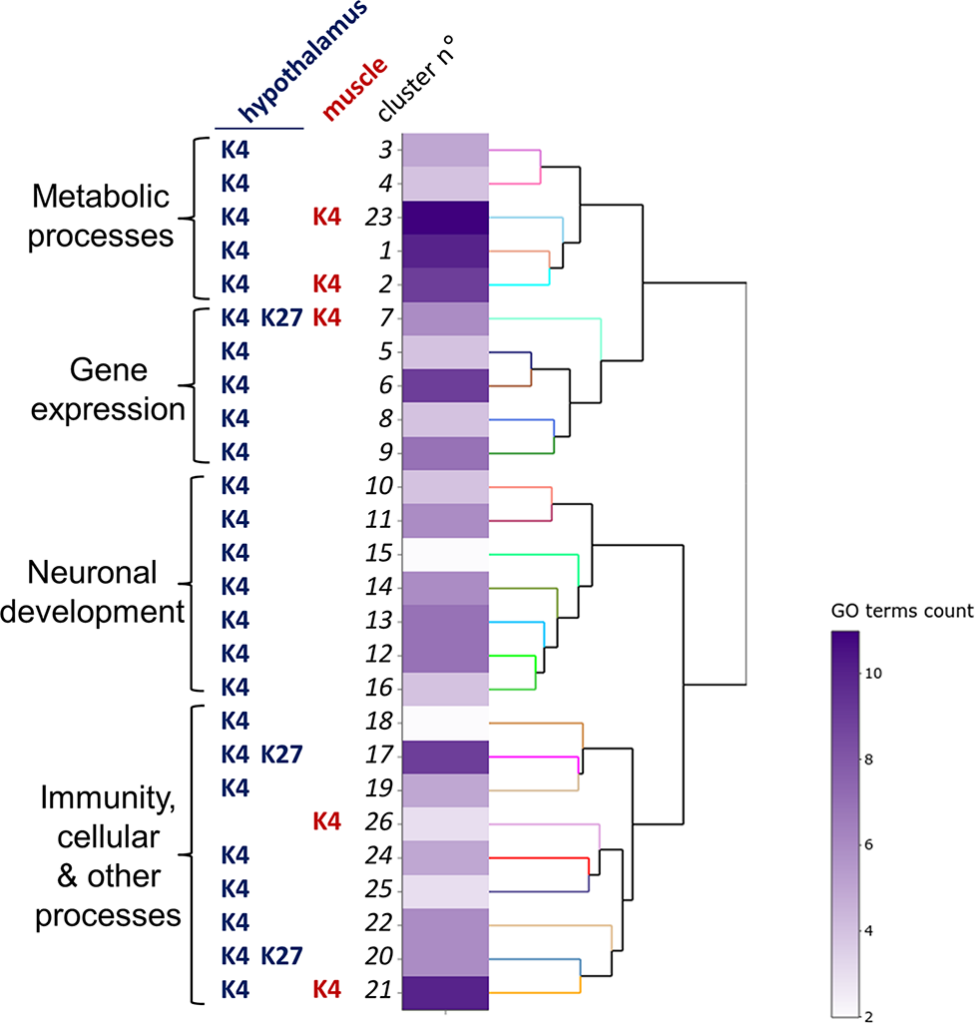
Functional analysis of hypothalamus and muscle genes bearing H3K4me3 and H3K27me3 differential peaks.The clustering heatmap plot of functional sets of gene ontology (GO) terms was obtained using ViSEAGO. From left to right are shown the major processes, the conditions defining the clusters (histone marks, tissues) defining the clusters, the cluster number, a heat map with the number of GO terms in each set and a dendrogram based on BMA semantic similarity distance and Ward’s clustering criterion.

To gain insights into the biological functions that may be affected by the presence of DP, we performed a functional analysis based on gene ontology (GO) analysis using the ViSEAGO tool (Brionne et al., 2019). Enrichment tests of GO terms associated with biological processes led to the identification of 154 enriched terms among the four list of genes (**Figure 2**, **Table 2**, and **Supplementary Figure S4** and **Table S3**). These terms were grouped into 26 clusters that could be further categorized into four major processes: neurodevelopment (36 GO terms), metabolism (39 GO terms), gene regulation (30 GO terms) and immunity, cellular and other processes (49 GO terms; **Supplementary Table S3**).

**Table 2:** Description of gene ontology (GO) clusters. For each cluster, the number of GO terms and the common ancestor term are shown

Cluster	GO Terms (total: 154)	Common ancestor term
1	10	Cellular metabolic process
2	9	Regulation of metabolic process
3	5	Regulation of macromolecule metabolic process
4	4	Regulation of macromolecule biosynthetic process
5	4	Regulation of nucleobase-containing compound metabolic process
6	9	Regulation of RNA biosynthetic process
7	6	Organic substance metabolic process
8	4	Nucleobase-containing compound metabolic process
9	7	Cellular biosynthetic process
10	4	Neuron projection morphogenesis
11	6	Anatomical structure morphogenesis
12	7	Gliogenesis
13	7	Cellular developmental process
14	6	System development
15	2	Cerebral cortex cell migration
16	4	Ensheathment of neurons
17	9	Multicellular organismal process
18	2	Positive regulation of muscle hypertrophy
19	5	Negative regulation of biological process
20	6	Microtubule cytosekeleton organization
21	10	Cellular process
22	6	Process
23	11	Biological regulation
24	5	Metal ion transport
25	3	Cytokine secretion
26	3	Protein localization to cell cortex

Six functional clusters comprised GO terms from different conditions. The 7^th^cluster, associated with gene expression regulation at the protein modification level, was defined by five GO terms corresponding to H3K4me3 DP (four for hypothalamus, one for muscle) and one GO term corresponding to hypothalamic H3K27me3 DP. However, none of the 144 genes defining this cluster were in common between conditions. Moreover, two clusters related to metabolic processes (2 and 23) and one related to cellular processes (21) resulted from GO terms enriched for H3K4me3 DP from both tissues. Finally, two clusters related to cellular processes (17 and 20) resulted from GO terms enriched for both H3K4me3 and H3K27me3 DP in the hypothalamus.

Interestingly, all clusters defining the neurodevelopmental function (10–16) were specific to the H3K4me3 mark and the hypothalamus, suggesting that this mark may contribute to the neurogenic processes of acclimation. This finding was confirmed by an analysis using the Ingenuity Pathway Analysis (IPA) software demonstrating that biological functions associated with hypothalamic H3K4m3 DP were mostly related to the nervous system development, including cellular development, growth and proliferation, as well as the cell-to-cell signaling (**Supplementary Table S4**).

### IPA Canonical Pathway Analysis Revealed an Impact of TM on H3K4me3 in Several Genes Related to the CRH Pathway and Previously Identified in the Postnatal Model

To gain further insights into the molecular mechanisms affected by TM, we investigated the canonical pathways using the IPA software and Fisher’s exact test. We focused on the hypothalamic H3K4me3 DP as it was the condition for which most DP were found. The most significantly enriched canonical pathways were mainly related to cellular growth and development such as AMPK signaling and *Oct4* (*Octamer-binding transcription factor 4*) function in embryonic cell, but were also related to the nervous system and behavior with calcium signaling, opioid signaling pathway, and synaptic long-term potential (**Table 3**). Interestingly, the CRH signaling pathway was also significantly enriched (p-value < 0.05; **Table 3** and **Supplementary Figure S5**). Molecules identified in this pathway included *ATF2* (Activating transcription factor 2), a transcriptional factor shown to be involved in the maintenance of the chromatin structure following embryonic stress in Drosophila (Seong et al., 2011), and *CRHR2* (*Corticotropin Releasing Hormone Receptor 2*), a receptor of the CRH hormone shown to be involved in determining later-life stress response of thermally-manipulated chicks (Cramer et al., 2015). In addition, the downstream target *BDNF* (*Brain Derived Neurotrophic Factor*; **Supplementary Figure S5**) was previously reported to be a major epigenetic target of postnatal thermal treatment in chicks (Yossifoff et al., 2008). To explore the impact of H3K4me3 changes of several members of the CRH pathway in TM animals, we measured the gene expression by RT-qPCR of several members of the pathway that contained a DP (*CRHR2*, *ATF2* and *MAPK11 – Mitogen-Activated Protein Kinase 11*; **Supplementary Figure S6**). We also investigated the expression of other key members of this pathway or downstream targets that did not contain a DP (*CRHR1*, *BDNF*, and *CREB1*–*CAMP Responsive Element Binding Protein 1*). Our results showed that none of these genes showed expression changes at a significance level of 0.05 even though *BDNF* and *CRHR1* showed a trend toward a decrease (both at P = 0.066; **Supplementary Figure S7**). Therefore, while TM appeared to significantly affect H3K4me3 marks for a subset of *CRH* pathway members, we could not demonstrate a clear link with gene expression in the biological replicates assayed.

**Table 3:** List of the canonical pathways identified by Ingenuity Pathway Analysis. The 25 significant pathways, with p-value < 0.05, are listed. The number and name of molecules participating in each pathway are shown in the “*n* =“ column

Ingenuity Canonical Pathways	-log(p-value)	*n*=	Molecules
AMPK Signaling	3.81	21	CHRNA5, ATF2, PPM1B, CAB39, PRKAR2B, PIK3CB, SMARCC1, FOXO6, RAB9B, RAB3A, PPARGC1A, CCND1, SMARCD3, PPP2R5D, CHRNB2, PRKAR1B, PPP2R2B, ELAVL1, PHF10, MAPK11, CHRM4
RAN Signaling	3.57	5	CSE1L, KPNA4, KPNA6, RAN, RANBP1
Role of Oct4 in Mammalian Embryonic Stem Cell Pluripotency	2.79	7	RARA, JARID2, BMI1, NR5A2, WWP2, CCNF, KDM5B
Geranylgeranyldiphosphate Biosynthesis	2.69	2	FDPS, GGPS1
Calcium Signaling	2.68	17	CHRNA5, ATF2, NFATC2, PRKAR2B, CAMK2D, MICU1, TRPC3, CACNA1B, ATP2B1, PPP3CA, ATP2B2, MCU, CHRNB2, PRKAR1B, CACNA2D2, TPM1, RYR2
CDK5 Signaling	2.47	11	PPP1R14C, KRAS, PRKAR2B, ADCY7, ITGB1, PPP2R5D, PRKAR1B, PPP2R2B, MAPK11, GNAL, MAPK6
Trans, Trans-farnesyl Diphosphate Biosynthesis	2.22	2	FDPS, GGPS1
BMP Signaling Pathway	1.83	8	KRAS, ATF2, PRKAR2B, PITX2, TLX2, PRKAR1B, MAPK11, SMURF1
Netrin Signaling	1.81	7	NFATC2, PRKAR2B, PRKAR1B, CACNA1B, PPP3CA, CACNA2D2, RYR2
GP6 Signaling Pathway	1.71	10	SCHIP1, COL4A6, LAMC3, PRKD3, KLF12, COL4A5, COL24A1, COL25A1, PIK3CB, PTK2
Cardiac Beta-Adrenergic Signaling	1.65	11	PPP1R14C, PDE4A, PRKAR2B, ADCY7, PDE4D, PPP2R5D, PRKAR1B, PPP2R2B, AKAP1, PDE10A, RYR2
Opioid Signaling Pathway	1.62	17	KRAS, ATF2, PRKAR2B, PRKD3, ADCY7, CLTC, CAMK2D, CACNA1B, PPP3CA, GNAL, RGS3, MAPK6, PRKAR1B, CACNA2D2, RGS4, GRK6, RYR2
Sonic Hedgehog Signaling	1.61	4	GLIS2, PRKAR2B, PRKAR1B, HHIP
Corticotropin Releasing Hormone Signaling	1.6	11	ATF2, GUCY1A2, PRKAR2B, PRKD3, UCN, ADCY7, PRKAR1B, CACNA1B, MAPK11, CACNA2D2, CRHR2
Synaptic Long Term Potentiation	1.59	9	PPP1R14C, KRAS, GRM2, ATF2, PRKAR2B, PRKD3, CAMK2D, PRKAR1B, PLCL2, PPP3CA
Dopamine-DARPP32 Feedback in cAMP Signaling	1.58	12	PPP1R14C, ATF2, GUCY1A2, PRKAR2B, PRKD3, ADCY7, PPP2R5D, PRKAR1B, PPP2R2B, PLCL2, PPP3CA, KCNJ10
cAMP-Mediated Signaling	1.55	16	GRM2, ATF2, PDE4A, CNGA4, PRKAR2B, ADCY7, CAMK2D, PPP3CA, GNAL, PDE10A, PDE4D, PRKAR1B, CHRM4, RGS4, AKAP1, DUSP4
RAR Activation	1.52	13	RARA, PRKAR2B, PRKD3, ADCY7, PIK3CB, SMARCC1, ALDH1A2, PPARGC1A, SMARCD3, PRKAR1B, PHF10, MAPK11, SRA1
Protein Kinase A Signaling	1.49	21	ATF2, TCF3, PDE4A, NFATC2, CNGA4, PRKAR2B, PRKD3, ADCY7, CAMK2D, PPP3CA, PTK2, PDE10A, ANAPC10, PPP1R14C, PTPRG, PDE4D, PRKAR1B, PLCL2, AKAP1, DUSP4, RYR2
Sperm Motility	1.48	9	PDE4A, GUCY1A2, CNGA4, PRKAR2B, PRKD3, PDE4D, PRKAR1B, PLCL2, PTK2
Regulation of Cellular Mechanics by Calpain Protease	1.46	6	KRAS, CNGA4, CCND1, ITGB1, TLN2, PTK2
Synaptic Long Term Depression	1.44	12	IGF1R, KRAS, GRM2, GUCY1A2, PRKD3, PPP2R5D, PPP2R2B, PLCL2, CACNA1B, CACNA2D2, GNAL, RYR2
ERK/MAPK Signaling	1.41	13	KRAS, ATF2, PRKAR2B, TLN2, PIK3CB, PTK2, HSPB2, PPP1R14C, ITGB1, PPP2R5D, PRKAR1B, PPP2R2B, DUSP4
Renin-Angiotensin Signaling	1.4	10	KRAS, ATF2, PRKAR2B, PRKD3, ADCY7, SHC2, PRKAR1B, MAPK11, PIK3CB, PTK2
Mitotic Roles of Polo-Like Kinase	1.32	5	STAG2, PPP2R5D, PPP2R2B, SMC3, ANAPC10

### Muscle Differential Peaks Did Not Localize Within De Genes Previously Identified

Prior to this study, a chicken transcriptome microarray (accession number GSE70756) was performed to compare *P. major* gene expression profiles of TM and C chickens reared at 21°C and under heat challenge condition at 32°C for 5 h (TM-HC and C-HC, respectively) (Loyau et al., 2016). Among the DE genes in the muscle, 28 were found to be DE between C and TM, 128 between C-HC and C, and 759 between TM-HC and TM. We investigated whether the DP gene list overlapped to these DE gene lists. To that end, the microarray data was reannotated with the Galgal5 annotation used in this study, leading to a similar list of 24, 127, and 724 DE genes included in the analysis for C vs. TM, C-HC vs. C, and TM-HC vs. TM comparisons, respectively (**Supplementary Table S5**). None of the 44 H3K4me3 and 17 H3K27me3 DP genes corresponded to DE genes from all comparisons (**Supplementary Table S4**). We also investigated the presence of DP in candidate genes that were shown to be DE by targeted RT-qPCR approach in the pectoral muscle of TM animals compared to C, including the *DIO3* (*Iodothyronine Deiodinase 3*) and *PGC1-α* (*PPARG Coactivator 1 alpha*) metabolic genes (Loyau et al., 2014). However, none of these DE genes contained a DP in the present analysis (data not shown).

## Discussion

Many studies investigated the impact of embryonic heat treatment in birds (for a review see Loyau et al., 2015) at the phenotypic, metabolic, and gene expression levels, but the molecular mechanisms underlying these changes were not fully understood. Recently, the hypothesis of an epigenetic contribution was raised by the identification of long-term gene expression effects including the alteration of transcript abundance of key epigenetic players (Loyau et al., 2016). Here, we provided the first demonstration of an epigenetic effect induced during embryogenesis in broiler chickens by the genome-wide mapping of two histone marks, H3K4me3 and H3K27me3, selected for their known function in epigenetic memory.

With only 61 DP for both marks, muscle chromatin of TM chickens displayed little changes compared to control animals at 35 days of age. This surprising result, given the large number of DE genes resulting from TM in muscle of heat-challenged broilers identified in a previous study (Loyau et al., 2016), is unlikely to be due to technical limitations given the very high sequencing depth employed in this study, above 74 M uniquely mapped reads for H3K27me3 instead of 40 M as recommended by ENCODE (Landt and Marinov, 2012) and 41 to 61 M uniquely mapped reads for H3K4me3 whereas ENCODE recommends 20 M for this mark in humans (**Supplementary Table S1**). The number of biological replicates (3) was also in agreement with ENCODE recommendations (> 2). Finally, the ChIP muscle preparation was optimized as shown in a previous study (David et al., 2017b) and led to the identification of a large number of peaks (**Table 1**) that were of expected shape and distribution (**Supplementary Table S1** and **Figures S1**, **S2**, and **S8**). Therefore, we are confident in concluding that TM had a limited impact on muscle H3K4me3 and H3K27me3 marks in our study. We previously showed in a transcriptome study based on the same *P. major* muscle tissue from the same Cobb 500 line of the same age and raised in the same experimental unit, that a large number of genes were DE in heat-challenged animals when they were TM. We hypothesized that some of these DE genes may bear epigenetic marks that may condition their response when animals were further exposed to a heat challenge in later life. Among the 61 genes that contained a DP identified in our study, none were found in the transcriptome data reannotated to match the current annotation. This suggests that both H3K4me3 and H3K27me3 marks are unlikely to be directly responsible for the DE genes resulting from TM previously observed in muscle. Whether or not other epigenetic marks or nonepigenetic mechanisms might be involved in long-term muscle TM response remains to be shown.

TM had a much stronger impact on both marks in the hypothalamus of 35-day-old chickens, with about 150 DP for H3K27me3 and 785 for H3K4me3. About 75% of H3K4me3 DP occurred at the 5’ end of genes, which is mainly explained by the sharp distribution of this mark around gene TSS. Nonetheless, H3K4me3 DP were underrepresented in intergenic regions, suggesting that TM is likely to preferentially impact H3K4me3 peaks possibly controlling gene expression. We confirmed the differential enrichment identified at four candidate loci by ChIP-qPCR in four biological replicates per condition, suggesting that the epigenetic impact of TM was reproducible within the flock. However, among the five DP genes tested for differential expression by qRT-PCR, none seemed to be altered in expression (**Supplementary Figures S4** and **S7**). Even though H3K4me3 is globally associated with transcriptional activity, many reports also showed that epigenetic marks, in particular when analyzed individually, are not always instructive of transcriptional status (Dong and Weng, 2013; Howe et al., 2017). Instead, TM-induced DP may also promote changes in expression in response to a stimulus (such as heat) rather than a constitutive change in gene expression that may be deleterious for development under normal conditions. For instance, a post-natal heat conditioning of 3-day-old chicks affected the expression of key factors such as *BDNF* an *DNMT3A* (Yossifoff et al., 2008), *EZH2* (Kisliouk et al., 2011), *HSP70* (Kisliouk et al., 2017), *CRH* (Cramer et al., 2015), and *Eif2b5* (Kisliouk et al., 2010) only during a 24-hour heat exposure at 10 days of age but not in control rearing conditions. To test this hypothesis, a new experiment would be required to measure the expression of DP genes in the hypothalamus during a heat challenge at 35 days of age as performed previously (Loyau et al., 2016).

The HSP70 family of chaperone proteins was shown to be involved in the cytoprotection of heat acclimated rats (Horowitz, 2017). In addition, the methylation level of a distal part of the HSP70 promoter was suggested to reflect heat-stress-related epigenetic memory in postnatally heat-conditioned chicks (Kisliouk et al., 2017). All *HSP* and *HSF* were checked for DP in all conditions. None appeared to display a differential peak with the exception of *HSPB2* for which a significantly higher peak of H3K4me3 signal was found in the hypothalamus. Despite not being a member of the HSP70 family, the small HSPB2 chaperone was recently shown to be associated with neuropathies when disregulated (Yu et al., 2018) and thus may play a role in neuron proliferation, a process shown to be important for acclimation (Matsuzaki et al., 2017).

To explore which biological functions may have been impacted by histone mark changes induced by TM, we performed a comprehensive GO analysis including all conditions (mark, tissue). No significantly enriched GO term was found for muscle H3K27me3, likely due to the very low number of DP genes (17) in that condition. Thirty-nine GO terms identified were related to metabolic processes. This is consistent with findings showing that TM was shown to affect the expression of several metabolic regulator genes in muscle (Loyau et al., 2014) and to the fact that heat acclimation is known to impact metabolic rate in several animal models (Collin et al., 2001; Shein et al., 2007; Piestun et al., 2009). Thirty-six GO terms were associated with neuronal development. IPA biological function analysis confirmed an impact of TM on H3K4me3 peaks localized at genes controlling neurodevelopmental functions. This finding is consistent with previous reports in rats showing that neurogenesis plays an important role in the establishment of heat acclimation in this species (Matsuzaki et al., 2009; Shido and Matsuzaki, 2015). Heat exposure of 5-week-old rats was notably shown to induce GABAergic and/or glutamatergic heat-responsive neurons in the preoptic area of the hypothalamus, influencing autonomic thermoregulation in the long term (Matsuzaki et al., 2017). Thirty GO terms were associated with the regulation of gene expression, which is consistent with a previous hypothesis suggesting that heat exposure during embryogenesis may impact the dynamics of chromatin architecture to allow access of the regulatory transcription machinery to favor efficient response of TM birds to heat (Loyau et al., 2016). Interestingly, one functional cluster was shared by both marks and tissues (excluding muscle H3K27me3, for which no GO term was found as stated above). This cluster is associated with gene regulation at the protein modification level (sialylation, peptidyl-methionine modification or ubiquitination) reinforcing the idea that chromatin structure is a key player for long-term acclimation (Loyau et al., 2016). Finally, 49 GO terms were associated with various cellular processes that notably involve immune responsein agreement with previous findings showing that, in the muscle, genes controlling immune or inflammatory responses were found be DE in TM broilers during heat stress in later life (Loyau et al., 2016). Additionally, in rats, heat acclimation was also shown to alter immune functions (Schneider and Zuhl, 2016). Altogether, TM affected the enrichment level of histone marks at a number of genes involved in several functions previously shown to be impacted by the TM or other heat acclimation models.

One interesting finding highlighted by the IPA H3K4me3 DP analysis was the identification, among others, of the CRH signaling pathway as significantly enriched (**Supplementary Figure S5**). The CRH pathway is involved in the neuroendocrine, autonomic, and behavioral stress responses (Kovács, 2013). Several key players of the CRH signaling pathway, including the ones cited below, were identified as bearing a H3K4me3 DP. Among them, *CRHR2* encodes one of the two downstream G protein-coupled receptors of CRH involved in the increase of intracellular cAMP levels (Inda et al., 2017). A downstream target in the pathways identified in our study is *MAPK11*, encoding one of the four p38 MAPKs which was shown to participate to heat acclimation in rats (Horowitz, 2014). A substrate for this enzyme is the transcriptional factor ATF2, and the *ATF2* gene also contained a DP. Remarkably, ATF2 was shown to be involved in heterochromatin formation and heterochromatin disruption following heat stress-induced activation (Seong et al., 2011). ATF2 belongs to the cAMP-responsive element (CRE) family that comprises the transcription factor CREB which target genes include *BDNF*. *BDNF* was reported more than a decade ago to be a key player in the thermal-experience-dependent plasticity of the hypothalamus in the postnatal acclimation model in chicks (Katz and Meiri, 2006). *BDNF* changes in expression were shown to be under the control of epigenetic alterations in the postnatal model (Yossifoff et al., 2008). *BDNF* was not part of the list of DP-containing genes but we tested to see if changes in the CRH pathway may influence its expression in the hypothalamus of TM animals. We therefore tested the gene expression of *CRHR2*, *ATF2*, *MAPK11* (DP genes) alongside the expression of *CRHR1*, *CREB1*, and *BDNF*. None of these genes appeared significantly misregulated, but *BDNF* showed a decrease in expression at the significance threshold of 0.1 (**Supplementary Figure S7**) that was replicated twice (data not shown). *CRHR1* also showed a decrease at the same significance threshold. More biological replicates might be necessary to verify this hypothesis. Nonetheless, it is worth mentioning that *BDNF* expression was shown to be increased by heat during postnatal conditioning (Katz and Meiri, 2006), and during a heat challenge following conditioning at 10 days of age (Yossifoff et al., 2008), but not after conditioning in absence of heat challenge. It would therefore be interesting to analyze *BDNF* expression, among other components of the CRH pathway, during or right after a heat exposure as was previously performed (Loyau et al., 2016). Finally, it is worth noting that we previously showed that the corticosterone (stress hormone) response seemed to be reduced in heat stressed TM chickens (vs. TM) compared to heat-stressed control chickens (vs. C) (Loyau et al., 2013). Given the interplay between corticosterone levels and *CRH* expression (Makino et al., 1994), it remains to be shown whether the changes in CRH signaling were induced directly during TM treatment and/or are a consequence of different stress response of TM animals during their lifetime until sampling.

In conclusion, this study provides the first line of evidence that embryonic TM in chicken involves lasting epigenetic changes in the hypothalamus that may contribute to the thermal acclimation of the animals.

## Methods

### Animals

Experiments were performed in accordance with the legislation governing the ethical treatment of birds and were approved by the French Ministry of Higher Education and the Val De Loire Animal Ethics Committee (Authorization N°APAFIS#4608-201603211212171v2). Cobb 500 eggs were supplied by Hendrix Genetics (Saint-Laurent-de-la-Plaine, France). The 500 eggs were incubated and hatched and animals were raised at the INRA UE1295 PEAT Poultry Experimental Facility (2018, https://doi.org/10.15454/1.5572326250887292E12). Half of the eggs were incubated in TM conditions (39.5°C for 12h/day and 65% RH during E7-16, the rest of the time at 37.8°C and 56% RH) and the other half was incubated in standard condition (37.8°C, 56% RH). The 120 TM chicks and 143 control chicks were hatched, monitored for cloacal temperature using a KIMO KTT-310 probe thermometer (KIMO, Montpon Ménestérol, France) and sexed by venting. Male chickens were raised in four pens (two for TM and two for C) and slaughtered for Pectoralis Major muscle and hypothalamus sampling at 35 days of age. Statistical analysis of temperature at hatching was performed using a Student test. Six animals were used for muscle ChIP-seq experiments (three TM and three C) and six animals were used for hypothalamus ChIP-seq experiments (three TM and three C). For ChIP-qPCR validations, hypothalamus samples from six male animals (three TM and three C; 35 days old) from the same experimental batch were sampled. For RT-qPCR analysis, hypothalamus samples from 10 other male animals (six TM and four C; 35 days old) from the same experimental batch were sampled.

### Chromatin Immunoprecipitation, Library Construction, and Sequencing

Muscle native chromatin immunoprecipitation (ChIP) was performed as previously described (David et al., 2017b). For hypothalamus samples, tissues were ground in a mortar using a pestle both cooled using liquid nitrogen. Ground powder was transferred in 1.5 ml TPX tube containing fixation buffer (1% FA in PBS with 1 X Complete^™^ protease inhibitors, PBS-C, Roche) for 5 min at room temperature under agitation. The reaction was stopped by adding Glycine to 0,125M final. The samples were centrifuged for 5 min at 3,000 g and washed twice using 1-ml ice-cold PBS-C. Cells were then centrifuged 5 min at 5,000 g and cellular pellets were resuspended in 300 µl sodium Dodecyl Sulphate (SDS) Lysis Buffer (50 mM Tris-HCl pH 8, 10 mM EDTA pH 8, 1% SDS, 10% glycerol, 1 X Complete^™^ protease inhibitors). Tubes were vortexed 20 s, incubated 10 min on ice and vortexed 20 s again. Lysed nuclei were sonicated using a Bioruptor (Diagenode, Denville, USA) set to high setting (30 s ON, 30 s OFF; 4 × 5 min). Chromatin immunoprecipitation was then performed as previously described (David et al., 2017b) using 5 µl of H3K27me3 antibody (07–449, batch #2506493, Merck-Millipore, Billerica, USA) or 5 µl of H3K4me3 antibody (07–473, batch #JBC188194 for hypothalamus and #2717639 for muscle, Merck-Millipore, Billerica, USA). The Sequencing libraries were prepared using the NEB Next Ultra II DNA Library Prep kit for Illumina (New England Biolabs, Ipswich, USA). Ten ng of H3K27me3 IP DNA, 10 ng of H3K4me3 IP DNA and 100 ng of input DNA were used. For hypothalamus, 10, 7, and 4 cycles of PCR amplification were performed, respectively; for muscle, 7, 8, and 4 cycles of PCR amplification were performed, respectively. Agencourt AMPure XP beads (Beckman Coulter, Brea, USA) were used for the 200-bp size selection of DNA fragments. Library concentration was measured by Qubit™ dsDNA HS Assay Kit (Invitrogen, Carlsbad, USA).

All samples were sequenced by the GenomEast genomic platform (IGBMC, Illkirch, France). Sequencing was realized in single-end read with 50-bp fragments by an Illumina Hi-Seq 4,000 sequencer.

### Bioinformatic and Biostatistic Analyses of Peaks

Sequencing quality was verified by a FASTQC analysis version 0.11.8 (Babraham bioinformatics). All reads were mapped to the version 5 of the chicken reference genome (Gallus gallus 5.0, GenBank assembly accession GCA_000002315.3) using Bowtie 2 version 2.2.6.2 (Langmead and Salzberg, 2012) with default options. Read duplicates were removed using SAMtools version 1.7 (Li et al., 2009). H3K27me3 broad peak detection for both tissues was realized using epic version 0.1.25 (https://github.com/biocore-ntnu/epic), a reimplementation of SICER (Xu et al., 2014) (options: –fragment-size 50–gaps-allowed 2–false-discovery-rate-cutoff 0.05). For hypothalamus samples, H3K4me3 peak detection was realized using PePr (Zhang et al., 2014) (version 1.1.14) and default settings (option: –peaktype = SHARP). For H3K4me3 peaks in muscle samples, PePr window size of peak detection was fixed to 240 bp (options: –peaktype = SHARP -w 240) as peaks were too broad using default settings in this tissue. A union between controls and TM peaks was performed independently for each tissue to include in the analysis peaks specific from each treatment and peaks common between treatments. Counts at union peak level were computed using featureCounts (Liao et al., 2014) version 1.6.2 (options -F SAF -s 0).

Differential peaks (DP) between control and TM samples were identified using edgeR package version 3.20.8 (McCarthy et al., 2012) for the four data sets (H3K27me3 and H3K4me3 from muscle and hypothalamus samples). Raw counts were normalized for library size and peak composition using trimmed mean of M-values method as recommended by Dillies et al. (2013). Descriptive and diagnostic graphs, like MDS-plot, permitted to detect a technological batch effect corresponding to library preparation and sequencing. A generalized linear model was fitted on normalized counts for each peak including batch and challenging factors (TM vs. C). A likelihood ratio test was performed to test the effect of the challenging factor. Raw p-value were adjusted for multiple testing using the Benjamini-Hochberg procedure to control False Discovery Rate (Benjamini and Hochberg, 1995). Peaks with an adjusted p-value less than 0.05 were considered as differential.

Annotation of DP and analysis of their distribution was made with the GenomeFeatures R Package version 0.99 (https://forgemia.inra.fr/aurelien.brionne/GenomeFeatures) for promoter (3 Kbp upstream and 500 bp downstream of TSS), flanking exons (5’UTR, 3’UTR), exons, introns, and downstream (1 Kbp from TES) features on both strands. A DP was considered within a gene when at least 1 bp of the peak overlapped to the gene, from the TSS until the transcriptional end site (TES). Peaks were visualized with Integrative Genome Viewer (Robinson et al., 2011). BigWig files were obtained from bam files using bamCoverage from deepTools suite (Ramírez et al., 2016) version 3.0.0. Normalization factors were calculated by performing a ratio between the bam file which have the smallest number of unique reads aligned per condition and the bam file of interest of the corresponding condition. Then, mean of BigWig files in each condition and each tissue was realized with bigWigMerge and bedGraphToBigWig (UCSC bioinformatic tools (Kent et al., 2010).

### Functional Analysis

Biological interpretations were carried out using the GO public database with the use of Biological Process (BP) category using ViSEAGO R package (Brionne et al., 2019). Associated Gene terms were retrieved from EntrezGene database (Sept. 2018) for *Gallus gallus* and experimental GO terms from orthologous genes were added. Enrichment tests were performed using exact Fisher’s test for each mark and tissue (four lists of genes). Enriched GO terms (p < 0.01) were grouped into functional clusters using hierarchical clustering based on Wang’s semantic similarity between GO terms respecting GO graph topology and Ward’s criterion. To go further in the interpretation, these functional clusters were grouped using hierarchical clustering based on BMA distance between sets of GO terms and Ward’s criterion.

Ingenuity Pathway Analysis (IPA, Ingenuity Systems, Mountain View, CA; version 377306M, content version 27216297) was used to explore canonical pathways. Core analysis, originally developed to analyze the impacted biological functions from expression data set, was used on identified DP genes with gene symbol and the associated adjusted p-value to discover the impacted molecular and cellular functions.

### ChiP-qPCR

Hypothalamus were mechanically homogenized in 1 ml of 0,1X fixation buffer in PBS with 1 X Complete™ protease inhibitors (Roche) using 5-mm stainless steel beads (Retsch) and a Mixer Mill MM 400 (Retsch, 1 min at 10 Hz). The rest of the experiment was performed as the ChIP-seq experiments previously described using H3K4me3 antibody (07–473, batch #2839113, Merck-Millipore, Billerica, USA). Immunoprecipitated DNA was eluated in 60 µl of TE buffer. A selection of putative candidates was obtained from ChIP-seq DP data. DP selection was based on the adjusted p-value (< 0.05) and a fold change >2 or <0.5. qPCR primers were designed in or around the DP coordinates with NCBI Primer blast (https://www.ncbi.nlm.nih.gov/tools/primer-blast/index.cgi?LINK_LOC=BlastHome) (**Supplementary Table S6**). The qPCR was performed as previously described (David et al., 2017b) on 2 µl of ChIP DNA or 1 µl of input DNA in triplicates with 5 µM of each primer and Takyon No ROX SYBR 2X MasterMix blue dTTP (Eurogentec, Liege, Belgium) following manufacturer’s instructions. Reactions were performed on a LightCycler^®^480 Instrument (Roche Diagnostics, Basel, Switzerland) using the following program: denaturation 5 min at 95°C, 50 amplification cycles (10 s at 95°C, 15 s at 60°C, and 15 s at 72°C), melting curve (5 s at 95°C, 1 min at 65°C, continuous at 95°C) and cooling. Enrichments were determined with the percent input method [100*2^ (adjusted input - Ct (IP)]. Statistical analyses of ChIP-qPCR were performed using R (version 3.5.1) by comparing TM (n = 3) and C (n = 3) enrichment using a Wilcoxon signed rank test with one-sided (alternative hypothesis as less) at the 0.05 level of significance.

### RNA Isolation, Reverse Transcription and Quantitative PCR for Gene Expression

Gene expression was evaluated by reverse transcription followed by real-time PCR as previously described. Total RNA was extracted from hypothalamus of biological replicates using AllPrep RNA/DNA Mini kit (Qiagen), according to the manufacturer’s instruction. RNA integrity was verified by electrophoresis on agarose gel. RNA was quantified on a Nanodrop ND-1000 UV-Vis Spectrophotometer.

cDNAs were synthesized from 2 µg of total RNA using the Superscript II enzyme (Invitrogen) and hexamer random primers (Promega) in 20 µl final volume, following the manufacturer’s instructions. Quantitative real-time PCR (qPCR) was carried out with Takyon qPCR Kits (Eurogentec) and using a LightCycler^®^480 Instrument II system (Roche) with 384-well plates (4TI-0382, 4titude).

Three technical replicates were performed for each sample and a standard curve protocol was used to evaluate gene expression. Primer sequences (**Supplementary Table S7**) were designed with NCBI Primer blast or Primer3Plus (http://www.bioinformatics.nl/cgi-bin/primer3plus/primer3plus.cgi) software. To assess the amplification of the correct cDNA fragments, every PCR product size was checked on a 2% agarose gel and sequenced by Sanger sequencing (Genewiz). Relative expression was normalized to the expression of three reference genes (selected among four reference genes tested): *GAPDH*, *POLR2E*, and *CytB* using geNorm software (Vandesompele et al., 2002). Statistical analyses of gene expression were performed using R (version 3.5.1) by comparing TM (n = 6) and C (n = 4) enrichment using a Wilcoxon signed rank test with two-sided at the 0.05 level of significance.

## Data Availability Statement

The datasets generated for this study can be found in the ChIP-seq data have been deposited in the ArrayExpress database at EMBL-EBI (www.ebi.ac.uk/arrayexpress) under accession number E-MTAB-7297 (“ChIP-seq of H3K4me3 and H3K27me3 histone marks in Pectoralis major muscle of 35 days-old control and thermally-manipulated chickens”) and E-MTAB-7300 (“ChIP-seq of H3K4me3 and H3K27me3 histone marks in the hypothalamus of 35 days-old control and thermally manipulated chickens”).

## Ethics Statement

The animal study was reviewed and approved by the French Ministry of Higher Education and the Val De Loire Animal Ethics Committee (Authorization N°APAFIS#4608-201603211212171v2).

## Author Contributions

VC, AC, and S-AD designed the experiments. VC, S-AD, AVC, TB, SC, and NC performed the experiments. VC, CG, BP, CH-A, AB, AVC, YB and S-AD analyzed the results. VC, AVC, CG, AB, and CH-A wrote the manuscript. All authors read and approved the final manuscript.

## Funding

Studies were made possible by the financial support of INRA Department Animal Physiology and Livestock Systems (PHASE), project STRESSEPIMARK. S-AD PhD work was cofunded by INRA-PHASE and Region Centre-Val de Loire. AVC and CG were funded by the ANR JCJC “QuailHeatE” Research Program (ANR-15-CE02-0009-01).

## Conflict of Interest

The authors declare that the research was conducted in the absence of any commercial or financial relationships that could be construed as a potential conflict of interest.

## References

[B1] Benjamini Y. Hochberg Y. (1995). Controlling the False Discovery Rate: a Practical and Powerful Approach to Multiple Testing. J. R. Stat. Soc Ser. B. 57 (1), 289–300.

[B2] Brionne A. Juanchich A. Hennequet-Antier C. (2019). ViSEAGO: a Bioconductor package for clustering biological functions using Gene Ontology and semantic similarity. BioData Min. 12, 16. 10.1186/s13040-019-0204-1 31406507 PMC6685253

[B3] Collin A. van Milgen J. Dubois S. Noblet J. (2001). Effect of high temperature on feeding behaviour and heat production in group-housed young pigs. Br. J. Nutr. 86 (1), 63–70. 10.1079/BJN2001356 11432766

[B4] Coustham V. Li P. Strange A. Lister C. Song J. Dean C. (2012).Quantitative modulation of polycomb silencing underlies natural variation in vernalization. Science 337 (6094), 584–587. 10.1126/science.1221881 22798408

[B5] Cramer T. Kisliouk T. Yeshurun S. Meiri N. (2015). The balance between stress resilience and vulnerability is regulated by corticotropin-releasing hormone during the critical postnatal period for sensory development. Dev. Neurobiol. 75, 842–853. 10.1002/dneu.22252 25447645

[B6] David S.-A. Mersch M. Foissac S. Collin A. Pitel F. Coustham V. (2017a).Genome-wide epigenetic studies in chicken: a review. Epigenomes 1, 20. 10.3390/epigenomes1030020

[B7] David S.-A. Piégu B. Hennequet-Antier C. Pannetier M. Aguirre-Lavin T. Crochet S. (2017b). An assessment of fixed and native chromatin preparation methods to study histone post-translational modifications at a whole genome scale in skeletal muscle tissue. Biol. Proced. Online 19. 10. 10.1186/s12575-017-0059-0 PMC5576305 28855851

[B8] Dillies M. A. Rau A. Aubert J. Hennequet-Antier C. Jeanmougin M. Servant N. (2013). A comprehensive evaluation of normalization methods for Illumina high-throughput RNA sequencing data analysis.Brief. Bioinform. 14 (6), 671–683. 10.1093/bib/bbs046 22988256

[B9] Dong X. Weng Z. (2013). The correlation between histone modifications and gene expression. Epigenomics 5 (2), 113–116. 10.2217/epi.13.13 23566087 PMC4230708

[B10] Fabrizio P. Garvis S. Palladino F. (2019). Histone methylation and memory of environmental stress. Cells 8 (4), E339. 10.3390/cells8040339 30974922 PMC6523599

[B11] Feil R. Fraga M. F. (2012). Epigenetics and the environment: emerging patterns and implications. Nat. Rev. Genet. 13, 97–109. 10.1038/nrg3142 22215131

[B12] Fresard L. Morisson M. Brun J. M. Collin A. Pain B. Minvielle F. (2013).Epigenetics and phenotypic variability: some interesting insights from birds.Genet. Sel. Evol. 45, 12. 10.1186/1297-9686-45-16 23758635 PMC3693910

[B13] Havenstein G. B. Ferket P. R. Qureshi M. A. (2003). Growth, livability, and feed conversion of 1957 versus 2001 broilers when fed representative 1957 and 2001 broiler diets. Poult. Sci. 82 (10), 1500–1508. 10.1093/ps/82.10.1500 14601725

[B14] Heijmans B. T. Tobi E. W. Stein A. D. Putter H. Blauw G. J. Susser E. S. (2008). Persistent epigenetic differences associated with prenatal exposure to famine in humans. Proc. Natl. Acad. Sci. U. S. A. 105, 17046–17049. 10.1073/pnas.0806560105 18955703 PMC2579375

[B15] Horowitz M. (2014). Heat acclimation, epigenetics, and cytoprotection memory.Compr. Physiol. 4 (1), 199–230. 10.1002/cphy.c130025 24692139

[B16] Horowitz M. (2017). Heat acclimation-mediated cross-tolerance: origins in within-life epigenetics? Front. Physiol. 8, 1–10. 10.3389/fphys.2017.00548 28804462 PMC5532440

[B17] Howe F. S. Fischl H. Murray S. C. Mellor J. (2017). Is H3K4me3 instructive for transcription activation? Bio Essays. 39 (1), 1–12. 10.1002/bies.201600095 28004446

[B18] Inda C. Armando N. G. dos Santos Claro P. A. Silberstein S. (2017). Endocrinology and the brain: corticotropin-releasing hormone signaling.Endocr. Connect. 6 (6), R99–R120. 10.1530/EC-17-0111 28710078 PMC5551434

[B19] Katz A. Meiri N. (2006). Brain-derived neurotrophic factor is critically involved in thermal-experience-dependent developmental plasticity. J. Neurosci. 26, 3899–3907. 10.1523/JNEUROSCI.0371-06.2006 16611805 PMC6673892

[B20] Kent W. J. Zweig A. S. Barber G. Hinrichs A. S. Karolchik D. (2010). Bigwig and Bigbed: enabling browsing of large distributed datasets. Bioinformatics. 26 (17), 2204–2207. 10.1093/bioinformatics/btq351 20639541 PMC2922891

[B21] Kisliouk T. Ziv M. Meiri N. (2010). Epigenetic control of translation regulation: Alterations in histone H3 lysine 9 post-translation modifications are correlated with the expression of the translation initiation factor 2B (Eif2b5) during thermal control establishment. Dev. Neurobiol. 70, 100–113. 10.1002/dneu.20763 19950192

[B22] Kisliouk T. Yosefi S. Meiri N. (2011). MiR-138 inhibits EZH2 methyltransferase expression and methylation of histone H3 at lysine 27, and affects thermotolerance acquisition. Eur. J. Neurosci. 33, 224–235. 10.1111/j.1460-9568.2010.07493.x 21070394

[B23] Kisliouk T. Cramer T. Meiri N. (2017). Methyl CpG level at distal part of heat-shock protein promoter HSP70 exhibits epigenetic memory for heat stress by modulating recruitment of POU2F1-associated nucleosome-remodeling deacetylase (NuRD) complex. J. Neurochem. 141, 358–372. 10.1111/jnc.14014 28278364

[B24] Kovács K. J. (2013). CRH: The link between hormonal-, metabolic- and behavioral responses to stress. J. Chem. Neuroanat. 54, 25–33. 10.1016/j.jchemneu.2013.05.003 23774011

[B25] Landt S. Marinov G. (2012). ChIP-seq guidelines and practices of the ENCODE and modENCODE consortia. Genome Res. 22 (9), 1813–1831. 10.1101/gr.136184.111 22955991 PMC3431496

[B26] Langmead B. Salzberg S. L. (2012). Fast gapped-read alignment with Bowtie 2 Nat. Methods 9,357–359. 10.1038/nmeth.1923 PMC3322381 22388286

[B27] Li H. Handsaker B. Wysoker A. Fennell T. Ruan J. Homer N. (2009). The Sequence Alignment/Map format and SAMtools. Bioinformatics. 25 (16), 2078–2079. 10.1093/bioinformatics/btp352 19505943 PMC2723002

[B28] Liao Y. Smyth G. K. Shi W. (2014). FeatureCounts: an efficient general purpose program for assigning sequence reads to genomic features. Bioinformatics. 30 (7), 923–930. 10.1093/bioinformatics/btt656 24227677

[B29] Loyau T. Berri C. Bedrani L. Métayer-Coustard S. Praud C. Duclos M. J. (2013). Thermal manipulation of the embryo modifies the physiology and body composition of broiler chickens reared in floor pens without affecting breast meat processing quality. J. Anim. Sci. 91, 3674–3685. 10.2527/jas2013-6445 23736053

[B30] Loyau T. Berri C. Crochet S. Cailleau-Audouin E. Sannier M. (2014). Thermal manipulation during embryogenesis has long-term effects on muscle and liver metabolism in fast-growing chickens. PloS One 9 (9), e105339. 10.1371/journal.pone.0105339 25180913 PMC4152147

[B31] Loyau T. Bedrani L. Berri C. Metayer-Coustard S. Praud C. Coustham V. (2015). Cyclic variations in incubation conditions induce adaptive responses to later heat exposure in chickens: a review. Animal 9 (1), 76–85. 10.1017/S1751731114001931 25118598

[B32] Loyau T. Hennequet-Antier C. Coustham V. Berri C. Leduc M. Crochet S. (2016). Thermal manipulation of the chicken embryo triggers differential gene expression in response to a later heat challenge. BMC Genomics 17, 329. 10.1186/s12864-016-2661-y 27142519 PMC4855354

[B33] Makino S. Gold P. W. Schulkin J. (1994). Corticosterone effects on corticotropin-releasing hormone mRNA in the central nucleus of the amygdala and the parvocellular region of the paraventricular nucleus of the hypothalamus. Brain Res. 640 (1–2), 105–112. 10.1016/0006-8993(94)91862-7 8004437

[B34] Matsuzaki K. Katakura M. Hara T. Li G. Hashimoto M. Shido O. (2009). Proliferation of neuronal progenitor cells and neuronal differentiation in the hypothalamus are enhanced in heat-acclimated rats. Pflugers Arch. Eur. J. Physiol. 458 (4), 661–673, 10.1007/s00424-009-0654-2 19252922

[B35] Matsuzaki K. Katakura M. Sugimoto N. Hara T. Hashimoto M. Shido O. (2017). Neural progenitor cell proliferation in the hypothalamus is involved in acquired heat tolerance in long-term heat-acclimated rats. PloS One 12, 1–16. 10.1371/journal.pone.0178787 PMC5476247 28628625

[B36] McCarthy D. J. Chen Y. Smyth G. K. (2012). Differential expression analysis of multifactor RNA-Seq experiments with respect to biological variation. Nucleic Acids Res. 40 (10), 4288–4297. 10.1093/nar/gks042 22287627 PMC3378882

[B37] Piestun Y. Shinder D. Ruzal M. Halevy O. Brake J. Yahav S. (2008). Thermal manipulations during broiler embryogenesis: effect on the acquisition of thermotolerance. Poult. Sci. 87, 1516–1525. 10.3382/ps.2008-00030 18648043

[B38] Piestun Y. Halevy O. Yahav S. (2009). Thermal manipulations of broiler embryos–the effect on thermoregulation and development during embryogenesis. Poultry Sci. 88 (12), 2677–2688. 10.3382/ps.2009-00231 19903968

[B39] Ramírez F. Ryan D. P. Grüning B. Bhardwaj V. Kilpert F. Richter A. S. (2016). Deeptools2: a next generation web server for deep-sequencing data analysis. Nucleic Acids Res. 44 (W1), W160–W165. 10.1093/nar/gkw257 27079975 PMC4987876

[B40] Robinson J. T. Thorvaldsdóttir H. Winckler W. Guttman M. Lander E. S. Getz G. (2011). Integrative genomics viewer. Nat. Biotechnol. 29 (1), 24–26. 10.1038/nbt.1754 21221095 PMC3346182

[B41] Schneider S. M. Zuhl M. N. (2016). HSP72 Up-regulation with heat acclimation.Temperature3,28–30. 10.1080/23328940.2016.1148525 PMC4861197 27227090

[B42] Seong K. H. Li D. Shimizu H. Nakamura R. Ishii S. (2011) .Inheritance of stress-induced, ATF-2-dependent epigenetic change. Cell 145, 1049–1061. 10.1016/j.cell.2011.05.029 21703449

[B43] Shein N. A. Horowitz M. Shohami E. (2007). Heat acclimation: a unique model of physiologically mediated global preconditioning against traumatic brain injury. Prog. Brain Res. 161, 353–363. 10.1016/S0079-6123(06)61025-X 17618990

[B44] Shido O. Matsuzaki K. (2015). Involvement of neurogenesis in the hypothalamic area in establishing long-term heat acclimation in rats.Temperature. 2 (3), 362–367. 10.1080/23328940.2015.1076591 PMC4843919 27227050

[B45] St-Pierre N. R. Cobanov B. Schnitkey G. (2003) .Economic Losses from Heat Stress by US Livestock Industries. J. Dairy Sci. 86, E52–E77. 10.3168/jds.S0022-0302(03)74040-5

[B46] Vandesompele J. De Preter K. Pattyn F. Poppe B. Van Roy N. De Paepe A., (2002). Accurate normalization of real-time quantitative RT-PCR data by geometric averaging of multiple internal control genes. Genome Biol. 3, RESEARCH0034. 10.1186/gb-2002-3-7-research0034 12184808 PMC126239

[B47] Xu S. Grullon S. Ge K. Peng W. (2014). Spatial clustering for identification of chip-enriched regions (SICER) to map regions of histone methylation patterns in embryonic stem cells. Methods Mol. Biol. 1150, 97–111. 10.1007/978-1-4939-0512-6_5 24743992 PMC4152844

[B48] Yahav S. McMurtry J. P. (2001). Thermotolerance acquisition in broiler chickens by temperature conditioning early in life - The effect of timing and ambient temperature. Poult. Sci. 80 (12), 1662–1666. 10.1093/ps/80.12.1662 11771878

[B49] Yossifoff M. Kisliouk T. Meiri N. (2008). Dynamic changes in DNA methylation during thermal control establishment affect CREB binding to the brain-derived neurotrophic factor promoter. Eur. J. Neurosci. 28, 2267–2277. 10.1111/j.1460-9568.2008.06532.x 19046370

[B50] Yu L. Petyuk V. A. Gaiteri C. Mostafavi S. Young-Pearse T. Shah R. C. (2018). Targeted brain proteomics uncover multiple pathways to Alzheimer’s dementia. Ann. Neurol. 84 (1), 78–88. 10.1002/ana.25266 29908079 PMC6119500

[B51] Zhang Y. Lin Y. H. Johnson T. D. Rozek L. S. Sartor M. A. (2014). PePr: a peak-calling prioritization pipeline to identify consistent or differential peaks from replicated ChIP-Seq data. Bioinformatics 30 (18), 2568–2575. 10.1093/bioinformatics/btu372 24894502 PMC4155259

