# Supplementary material for: Thermal Manipulation During Embryogenesis Impacts H3K4me3 and H3K27me3 Histone Marks in Chicken Hypothalamus
Source: Front Genet. 2019 Nov 26;10:1207. doi: 10.3389/fgene.2019.01207 (PMC6889634; doi:10.3389/fgene.2019.01207)
Supplement: Supplementary file 1 [file DataSheet_1.pdf]

Thermal manipulation during embryogenesis impacts H3K4me3 and H3K27me3 histone marks in chicken hypothalamus

Sarah-Anne David[1†], Anaïs Vitorino Carvalho[1†], Coralie Gimonnet[1], Aurélien Brionne[1], Christelle Hennequet-Antier[1], Benoît Piégu[2], Sabine Crochet[1], Nathalie Couroussé[1], Thierry Bordeau[1], Yves Bigot[2], Anne Collin[1] and Vincent Coustham[1]*

[1]BOA, INRA, Université de Tours, 37380, Nouzilly, France
[2]PRC, CNRS, IFCE, INRA, Université de Tours, 37380, Nouzilly, France

[†]These authors contributed equally to this work
*Corresponding author

Supplementary Figures

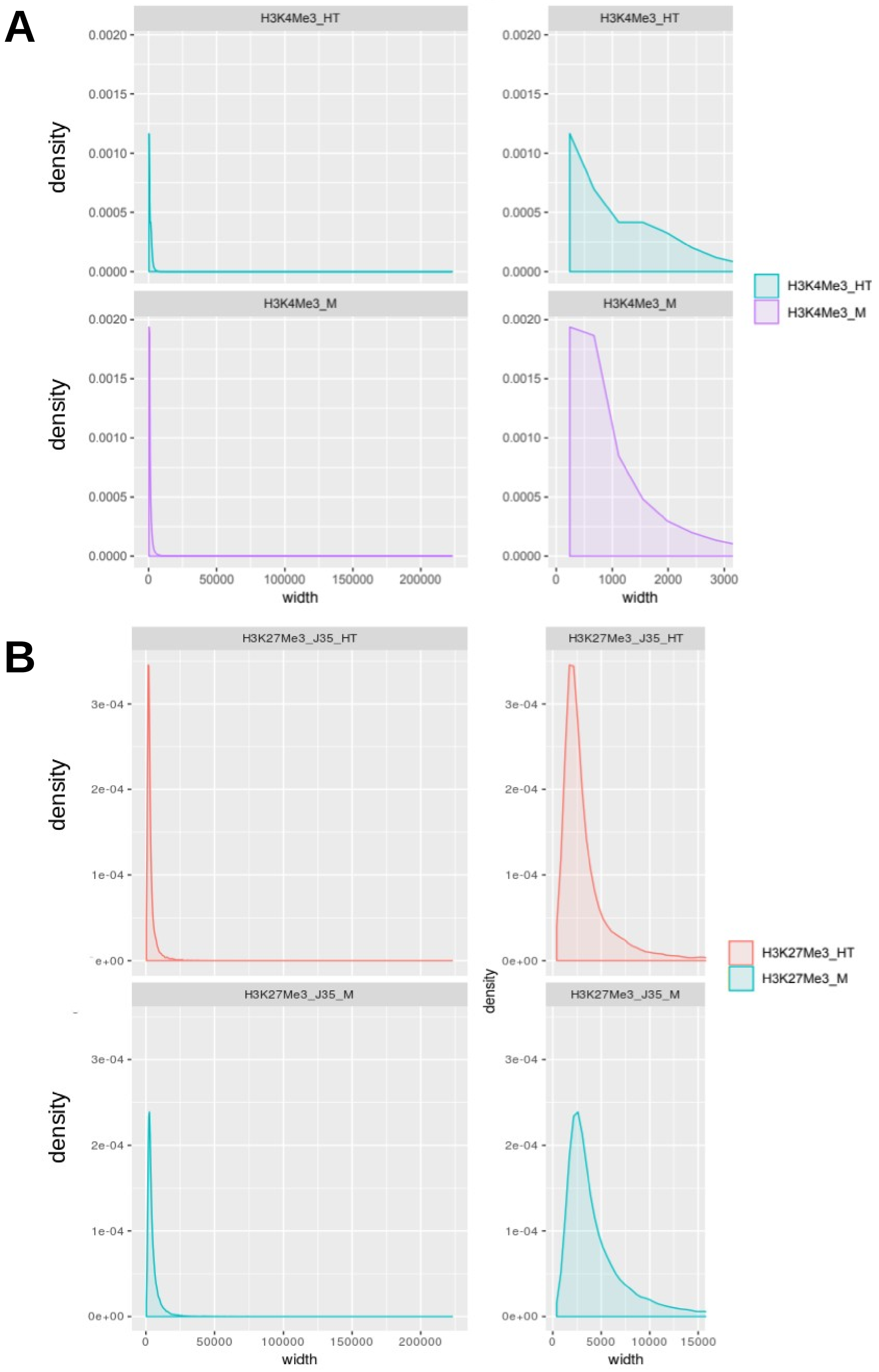

Fig. S1: Peak sizes. Density plots of (**A**) H3K4me3 and (**B**) H3K27me3 peaks width. Columns on the right correspond to a zoom on the density peaks. HT: hypothalamus; M: *Pectoralis major* muscle.

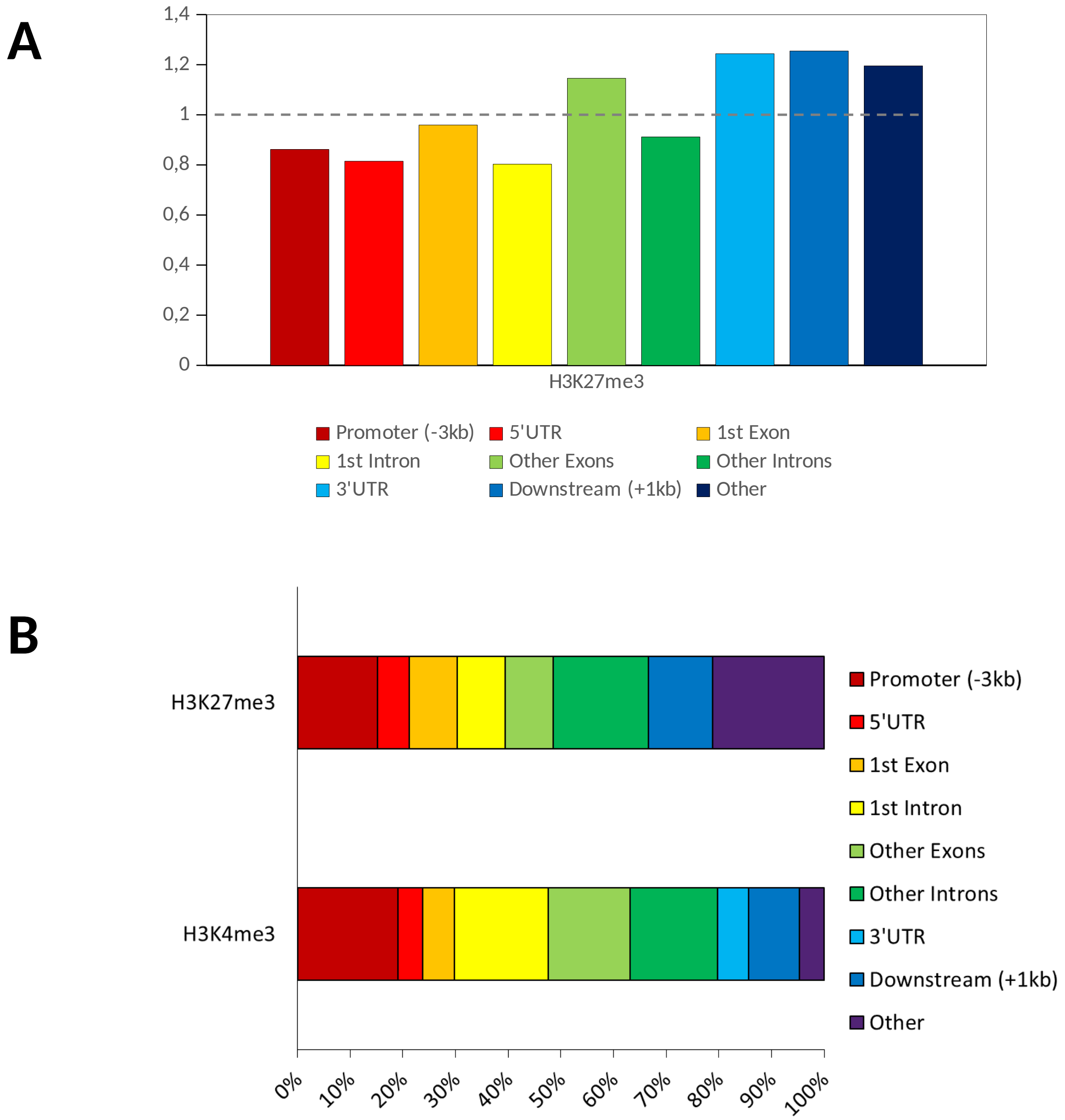

Fig. S2: Analysis of DP distribution. (A) Ratio of hypothalamus H3K27me3 DP/all analyzed peaks. (B) Distribution of muscle DP.

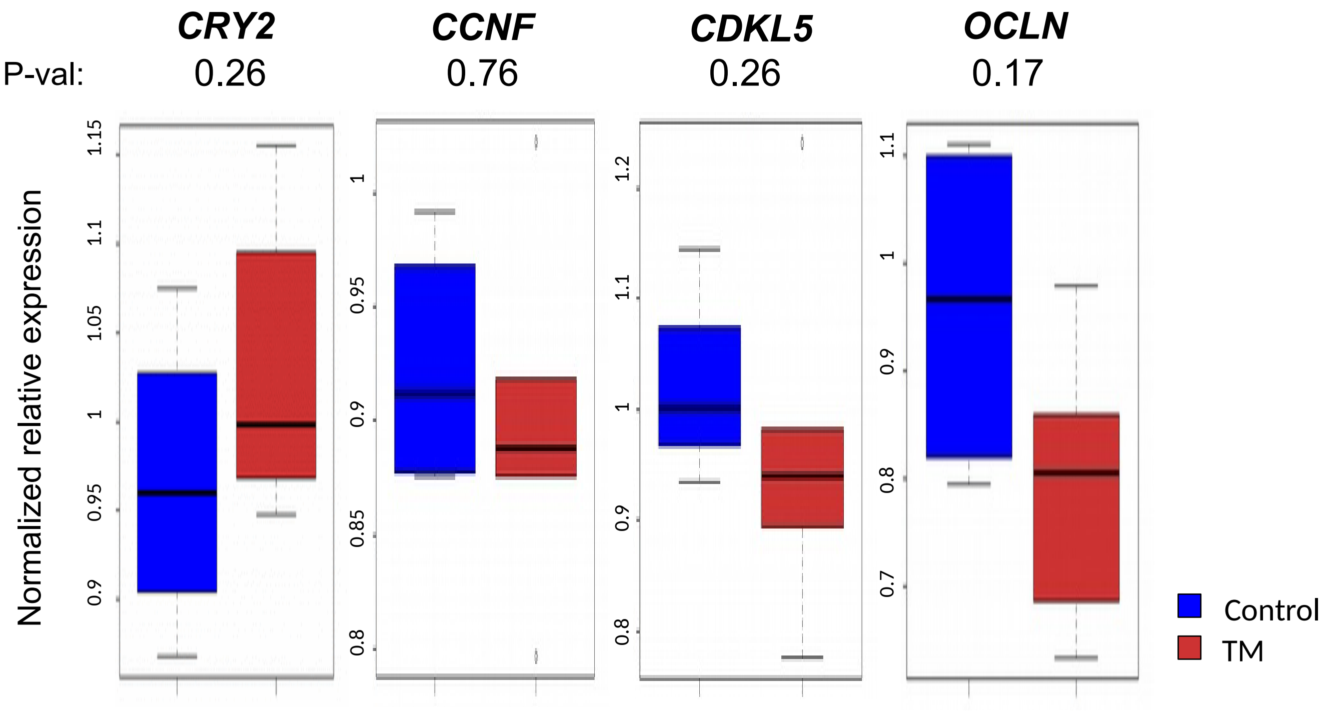

Fig. S3: Relative expression of candidate genes (*CRY2*, *CCNF*, *CDKL5* and *OCLN*) that contained a differential H3K4me3 peak. Control replicates are shown in blue, TM in red. Box boundaries represent the first and third quartiles. The median is indicated by the bold horizontal line dividing the interquartile range. Upper and lower ticks indicate the 10th and 90th percentiles.

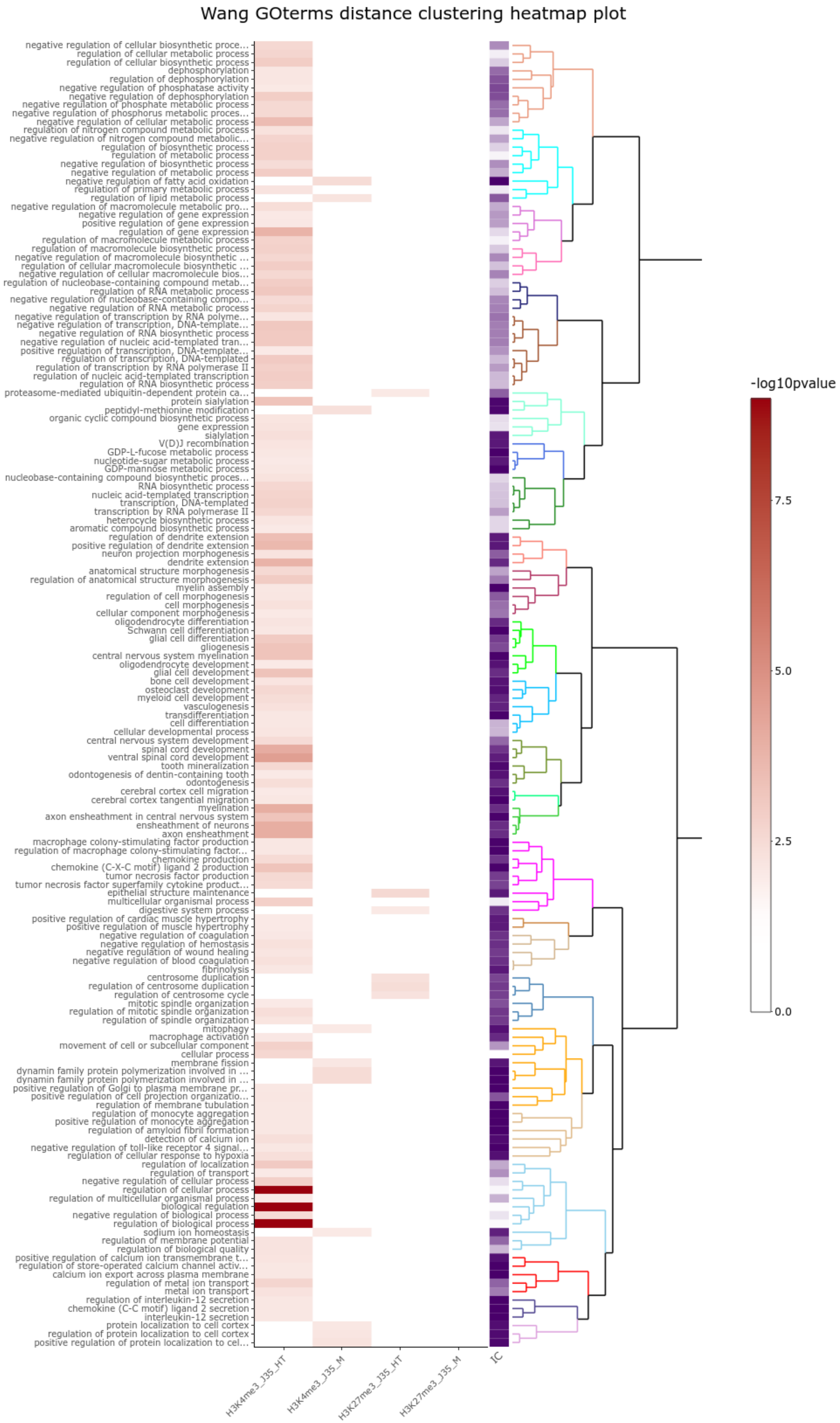

Wang GOterms distance clustering heatmap plot
negative regulation of cellular biosynthetic proce...
regulation of cellular metabolic process
regulation of cellular biosynthetic process
dephosphorylation
regulation of dephosphorylation
negative regulation of phosphatase activity
negative regulation of dephosphorylation
negative regulation of phosphate metabolic process
negative regulation of phosphorus metabolic proces...
negative regulation of cellular metabolic process
regulation of nitrogen compound metabolic process
negative regulation of nitrogen compound metabolic...
regulation of biosynthetic process
regulation of metabolic process
negative regulation of biosynthetic process
negative regulation of metabolic process
negative regulation of fatty acid oxidation
regulation of primary metabolic process
regulation of lipid metabolic process
negative regulation of macromolecule metabolic pro...
negative regulation of gene expression
positive regulation of gene expression
regulation of gene expression
regulation of macromolecule metabolic process
regulation of macromolecule biosynthetic process
negative regulation of macromolecule biosynthetic ...
regulation of cellular macromolecule biosynthetic ...
negative regulation of cellular macromolecule bios...
regulation of nucleobase-containing compound metab...
regulation of RNA metabolic process
negative regulation of nucleobase-containing compo...
negative regulation of RNA metabolic process
negative regulation of transcription by RNA polyme...
negative regulation of transcription, DNA-template...
negative regulation of RNA biosynthetic process
negative regulation of nucleic acid-templated tran...
positive regulation of transcription, DNA-template...
regulation of transcription, DNA-templated
regulation of transcription by RNA polymerase II
regulation of nucleic acid-templated transcription
regulation of RNA biosynthetic process
proteasome-mediated ubiquitin-dependent protein ca...
protein sialylation
peptidyl-methionine modification
organic cyclic compound biosynthetic process
gene expression
sialylation
V(D)J recombination
GDP-L-fucose metabolic process
nucleotide-sugar metabolic process
GDP-mannose metabolic process
nucleobase-containing compound biosynthetic proces...
RNA biosynthetic process
nucleic acid-templated transcription
transcription, DNA-templated
transcription by RNA polymerase II
heterocycle biosynthetic process
aromatic compound biosynthetic process
regulation of dendrite extension
positive regulation of dendrite extension
neuron projection morphogenesis
dendrite extension
anatomical structure morphogenesis
regulation of anatomical structure morphogenesis
myelin assembly
regulation of cell morphogenesis
cell morphogenesis
cellular component morphogenesis
oligodendrocyte differentiation
Schwann cell differentiation
glial cell differentiation
gliogenesis
central nervous system myelination
oligodendrocyte development
glial cell development
bone cell development
osteoclast development
myeloid cell development
vasculogenesis
transdifferentiation
cell differentiation
cellular developmental process
central nervous system development
spinal cord development
ventral spinal cord development
tooth mineralization
odontogenesis of dentin-containing tooth
odontogenesis
cerebral cortex cell migration
cerebral cortex tangential migration
myelination
axon ensheathment in central nervous system
ensheathment of neurons
axon ensheathment
macrophage colony-stimulating factor production
regulation of macrophage colony-stimulating factor...
chemokine production
chemokine (C-X-C motif) ligand 2 production
tumor necrosis factor production
tumor necrosis factor superfamily cytokine product...
epithelial structure maintenance
multicellular organismal process
digestive system process
positive regulation of cardiac muscle hypertrophy
positive regulation of muscle hypertrophy
negative regulation of coagulation
negative regulation of hemostasis
negative regulation of wound healing
negative regulation of blood coagulation
fibrinolysis
centrosome duplication
regulation of centrosome duplication
regulation of centrosome cycle
mitotic spindle organization
regulation of mitotic spindle organization
regulation of spindle organization
mitophagy
macrophage activation
movement of cell or subcellular component
cellular process
membrane fission
dynamin family protein polymerization involved in ...
dynamin family protein polymerization involved in ...
positive regulation of Golgi to plasma membrane pr...
positive regulation of cell projection organizatio...
regulation of membrane tubulation
regulation of monocyte aggregation
positive regulation of monocyte aggregation
regulation of amyloid fibril formation
detection of calcium ion
negative regulation of toll-like receptor 4 signal...
regulation of cellular response to hypoxia
regulation of localization
regulation of transport
negative regulation of cellular process
regulation of cellular process
regulation of multicellular organismal process
biological regulation
negative regulation of biological process
regulation of biological process
sodium ion homeostasis
regulation of membrane potential
regulation of biological quality
positive regulation of calcium ion transmembrane t...
regulation of store-operated calcium channel activ...
calcium ion export across plasma membrane
regulation of metal ion transport
metal ion transport
regulation of interleukin-12 secretion
chemokine (C-C motif) ligand 2 secretion
interleukin-12 secretion
protein localization to cell cortex
regulation of protein localization to cell cortex
positive regulation of protein localization to cel...
H3K4me3_J35_HT
H3K4me3_J35_M
H3K27me3_J35_HT
H3K27me3_J35_M
IC
-log10pvalue
7.5
5.0
2.5
0.0

Fig. S4: Functional analysis of hypothalamus and muscle genes bearing H3K4me3 and H3K27me3 differential peaks using a clustering heatmap plot from the ViSEAGO package. From left to right: heatmap of -log10(p-value) of GO terms from functional enrichment tests for each mark and tissue, information content (IC) and dendrogram based on Wang's semantic similarity distance and Ward's clustering criterion. ViSEAGO package is available at https://bioconductor.org/packages/ViSEAGO

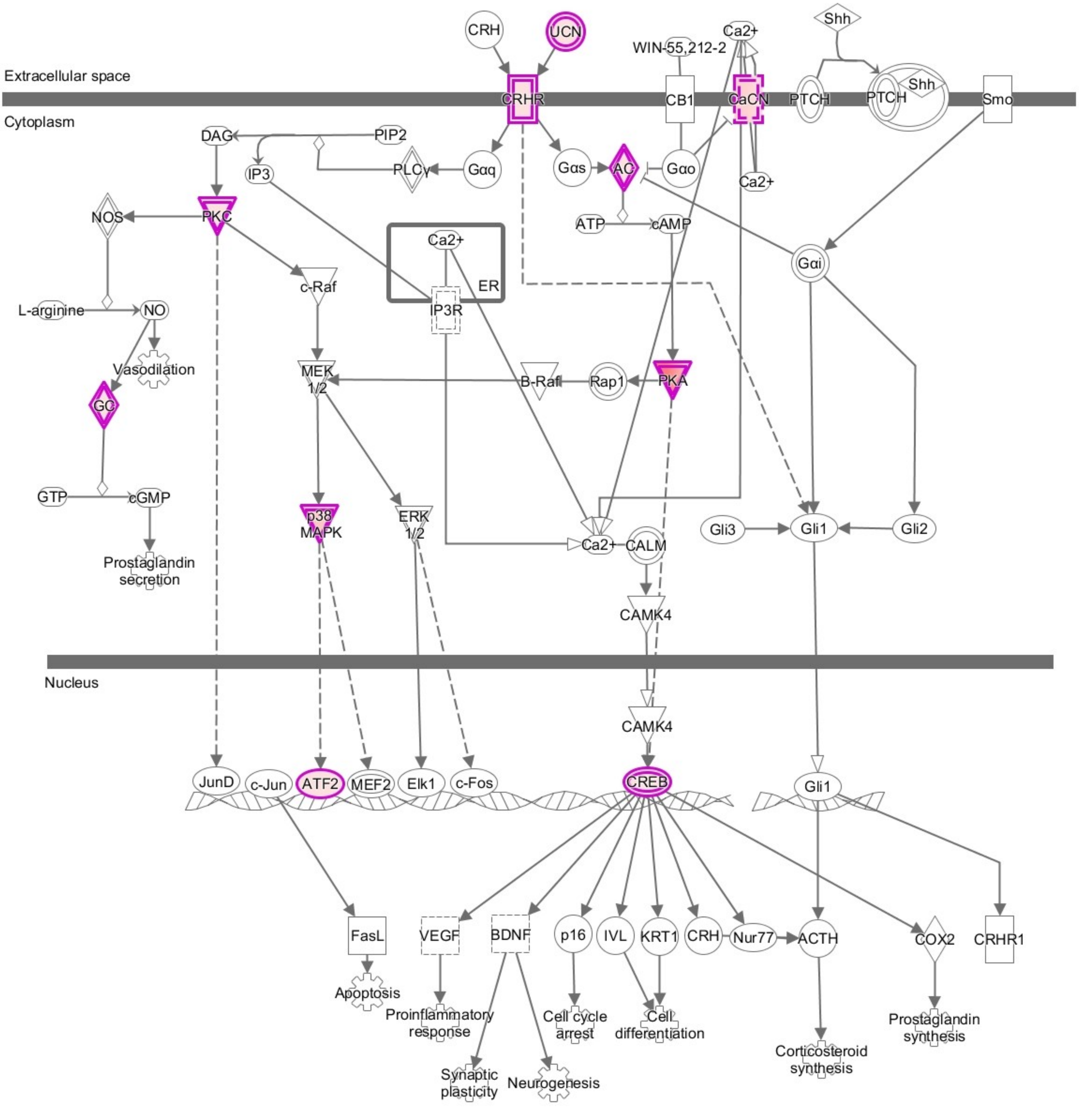

© 2000-2018 QIAGEN. All rights reserved.

Fig. S5: IPA canonical pathway of CRH signalling. Factors underlined in purple correspond to candidates containing DP identified in this study. Pink colouring correspond to an increase in TM condition. Double lines correspond to complexes. Triangles correspond to kinases, ovals to transcription factors, upright rectangles to ion channels, diamond shapes to enzymes, dashed squares to growth factors. Plain arrows correspond to activation/causation, dashed arrows to an indirect interaction. IPA legend can be found at https://mbio.asm.org/content/mbio/suppl/2014/12/11/mBio.02113-14.DCSupplemental/mbo006142092st4.pdf

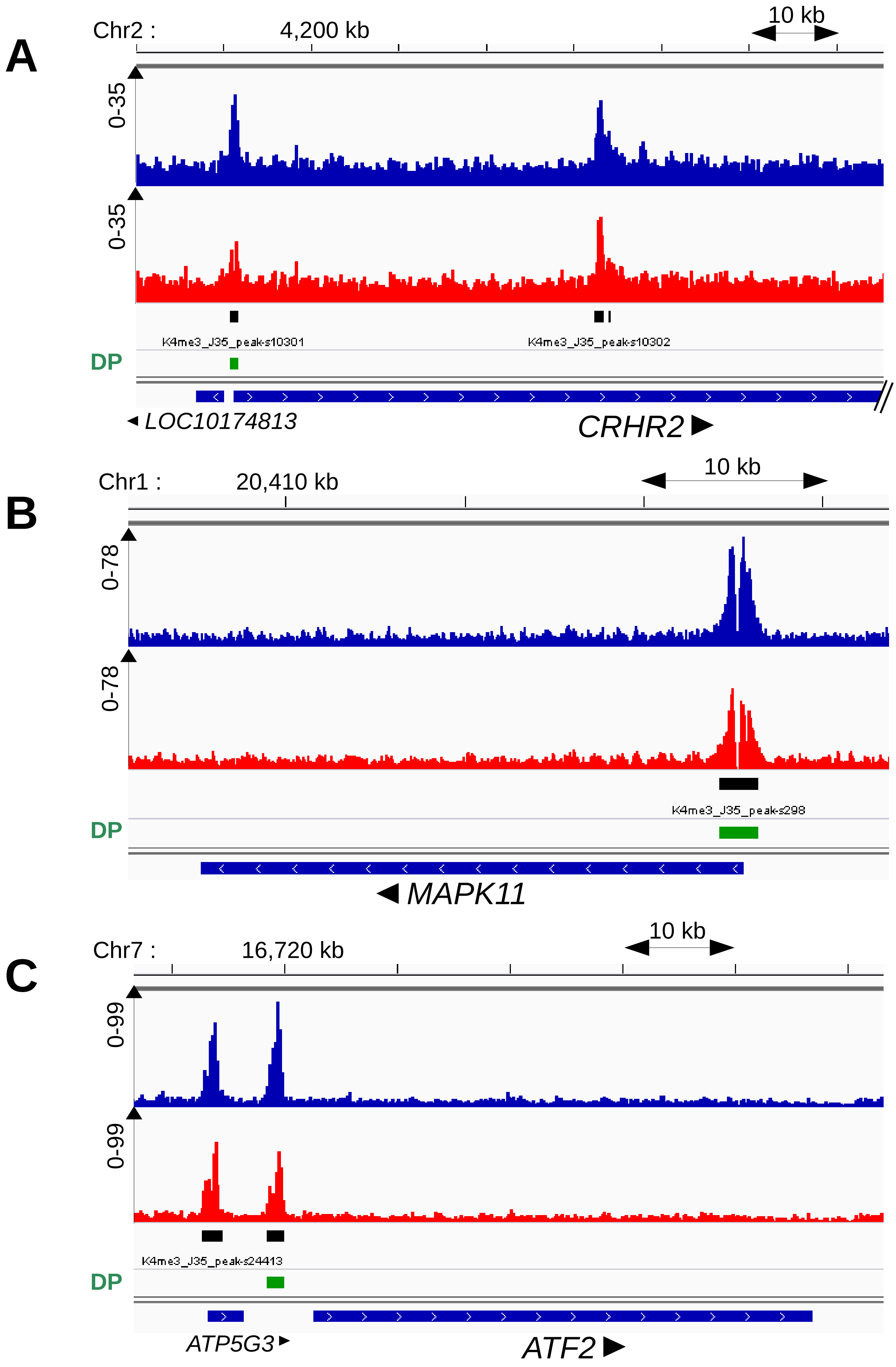

Fig. S6: Chromosome snapshots of hypothalamus H3K4me3 ChIP-seq data at (A) *CRHR2*, (B) *MAPK11* and (C) *ATF2* loci using Integrative Genomics viewer (IGV; Broad Institute). Tracks correspond to normalized and averaged BigWig data. Signal from H3K4me3 (blue for C, red for TM) ChIP-seq is shown. Scales have been grouped for each region using the IGV group autoscale option. Black boxes below the tracks indicate H3K4me3 peak coordinates used in the differential analysis. Green boxes indicate differential peaks (DP).

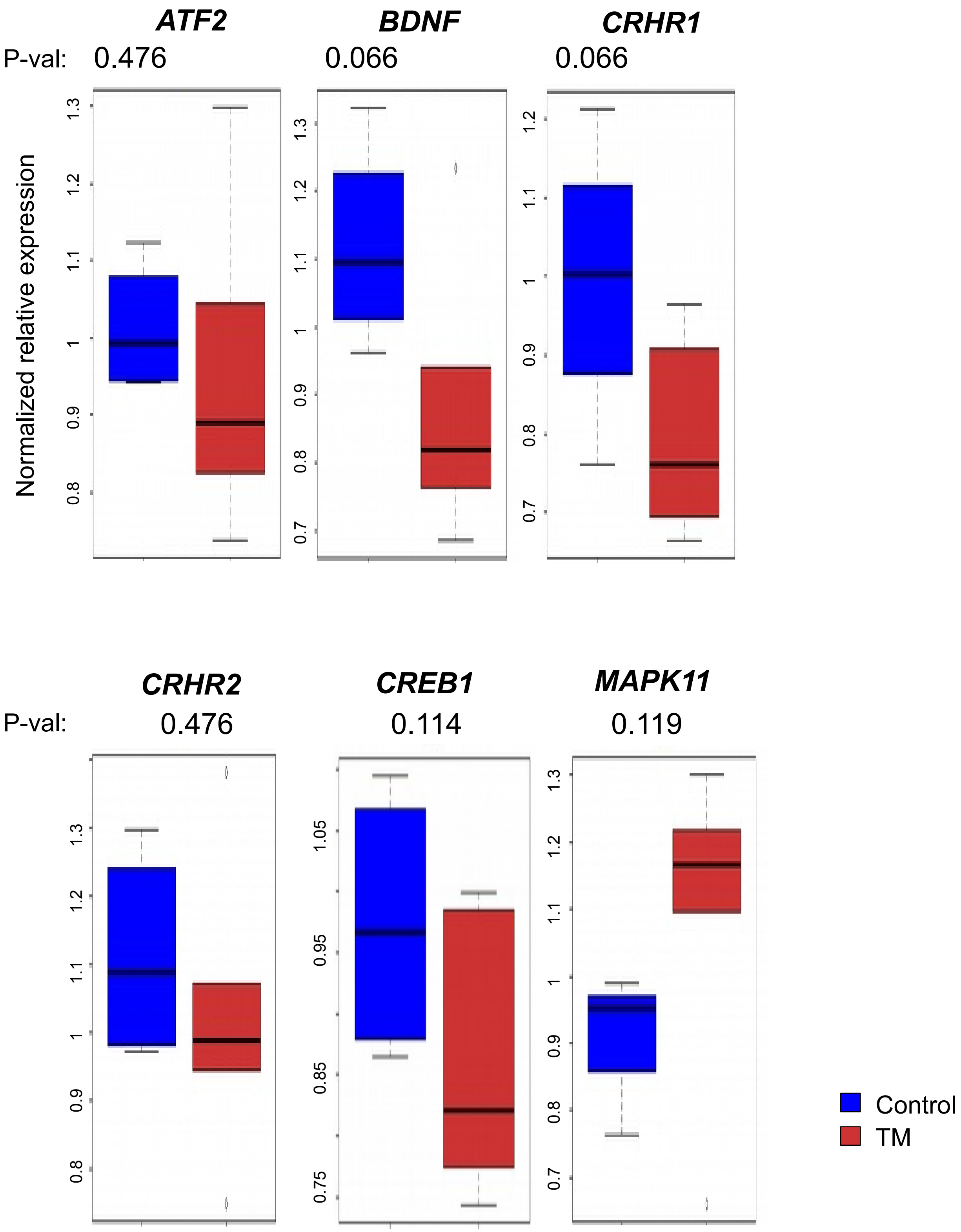

Fig. S7: Relative expression of candidate genes (*ATF2*, *BDNF*, *CRHR1, CRHR2, CREB1* and *MAPK11*) from the CRH signalling pathway. Control replicates are shown in blue, TM in red. Box boundaries represent the first and third quartiles. The median is indicated by the bold horizontal line dividing the interquartile range. Upper and lower ticks indicate the 10th and 90th percentiles. Outliers are indicate by circles.

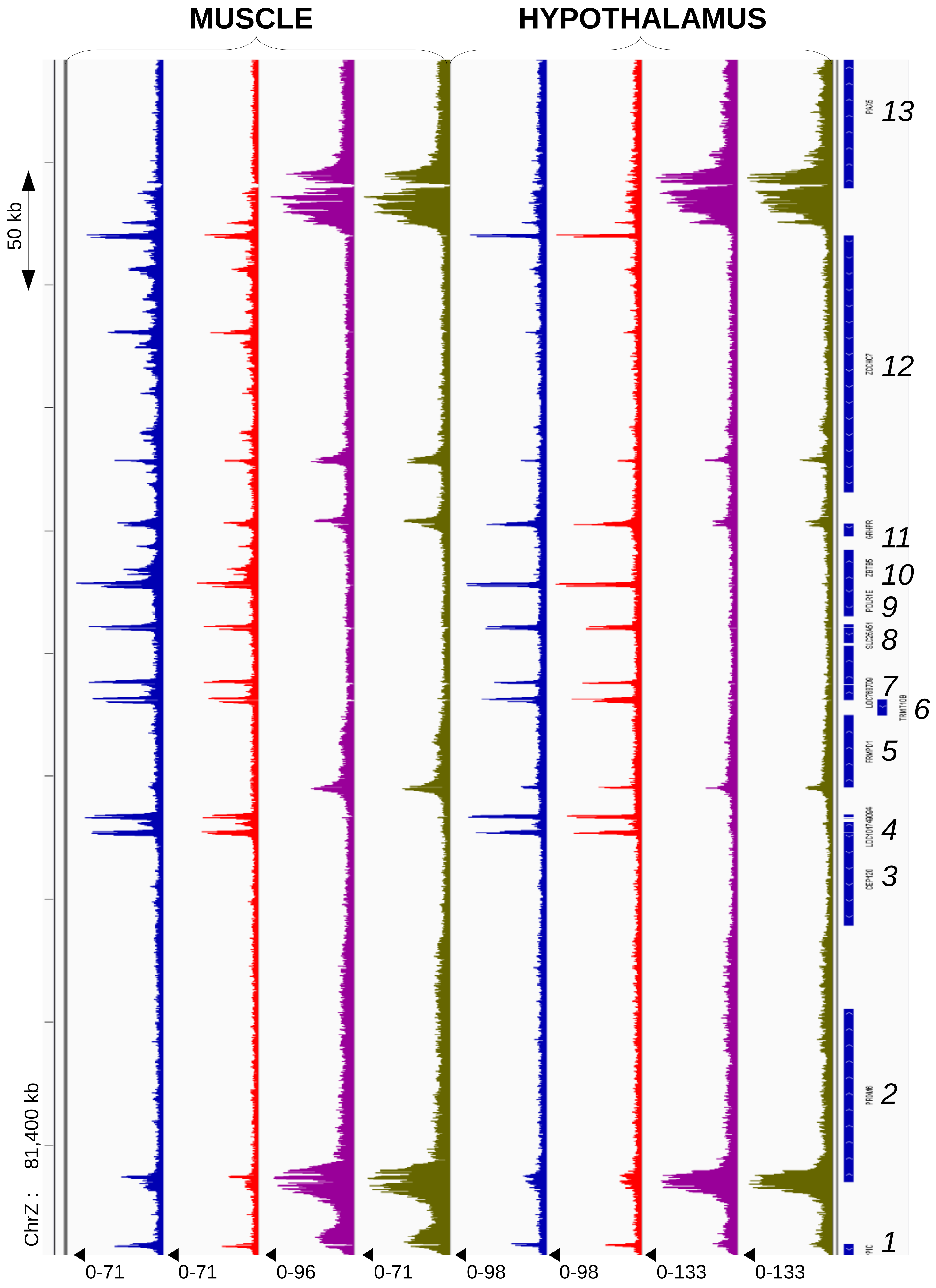

MUSCLE
HYPOTHALAMUS
50 kb
ChrZ : 81,400 kb
0-71
0-71
0-96
0-71
0-98
0-98
0-133
0-133
PAX5
13
ZCCHC7
12
GRHPR
11
ZBTB5
10
POLR1E
9
SLC25A51
8
LOC768709
7
TRMT10B
6
FRMPD1
5
LOC101749094
4
CEP120
3
PROM6
2
1

Fig. S8: Chromosome Z snapshot of muscle and hypothalamus H3K4me3 and H3K27me3 ChIP-seq data (chrZ:81,341,460-81,857,046) obtained using Integrative Genomics viewer (IGV; Broad Institute). Tracks correspond to normalized and averaged BigWig data. Signal from H3K4me3 (blue for C, red for TM) and H3K27me3 (purple for C, brown for TM) ChIP-seq is shown. Muscle tracks are shown on the left, hypothalamus tracks on the right. Scales have been grouped for each mark and tissue using the IGV group autoscale option. The plain blue boxes below the tracks indicate genes, correspondence is as following: *1: PPIC; 2: PRDM6; 3: CEP120; 4: LOC101749094; 5: FRMPD1; 6: TRMT10B; 7: LOC768709; 8: SLC25A51; 9: POLR1E; 10: ZBTB5; 11: GRHPR; 12: ZCCHC7; 13: PAX5*. In this region, only one H3K4me3 differential peak is present at the *LOC768709* promoter.
